# Importance of Characteristic Features and Their Form for Data Exploration

**DOI:** 10.3390/e26050404

**Published:** 2024-05-06

**Authors:** Urszula Stańczyk, Beata Zielosko, Grzegorz Baron

**Affiliations:** 1 Department of Computer Graphics, Vision and Digital Systems, Silesian University of Technology, Akademicka 2A, 44-100 Gliwice, Poland; grzegorz.baron@pols.pl; 2 Institute of Computer Science, University of Silesia in Katowice, Bȩdzińska 39, 41-200 Sosnowiec, Poland; beata.zielosko@us.edu.pl

**Keywords:** relevance, ranking, discretisation, attribute domain, pattern recognition, stylometry, 68T10, 68T30, 68T37, 68U35

## Abstract

The nature of the input features is one of the key factors indicating what kind of tools, methods, or approaches can be used in a knowledge discovery process. Depending on the characteristics of the available attributes, some techniques could lead to unsatisfactory performance or even may not proceed at all without additional preprocessing steps. The types of variables and their domains affect performance. Any changes to their form can influence it as well, or even enable some learners. On the other hand, the relevance of features for a task constitutes another element with a noticeable impact on data exploration. The importance of attributes can be estimated through the application of mechanisms belonging to the feature selection and reduction area, such as rankings. In the described research framework, the data form was conditioned on relevance by the proposed procedure of gradual discretisation controlled by a ranking of attributes. Supervised and unsupervised discretisation methods were employed to the datasets from the stylometric domain and the task of binary authorship attribution. For the selected classifiers, extensive tests were performed and they indicated many cases of enhanced prediction for partially discretised datasets.

## 1. Introduction

The knowledge discovery in databases (KDD) process refers to discovering useful knowledge or patterns from databases [1]. It encompasses a range of techniques and methodologies from various fields, such as machine learning, data mining, statistical analysis, and use of database systems [2]. The KDD process involves several stages, including data selection, preprocessing, transformation, mining, evaluation, and interpretation of results. It often begins with identifying relevant data sources, followed by cleaning and preprocessing the data to handle noise, missing values, and inconsistencies. Subsequently, various data mining techniques are applied to discover patterns, associations, or clusters within the input data [3]. The discovered patterns are then evaluated to assess their significance and reliability. Finally, the results are presented in a meaningful way to facilitate decision-making and knowledge utilisation and interpretation.

Feature selection [4] plays one of the key roles in the KDD process, more specifically in the data preparation stage. The objective is to identify significant attributes from the entire set of available variables, while at the same time preserving the descriptive and representative qualities of the original set of the input features [5]. One of the means to assess the quality of selected features is the construction of a ranking of attributes [6]. It enables ordering of variables from the most to the least important, based on some adopted criterion. Feature ranking is also referred to as feature weighting. It encompasses the evaluation of individual attributes through the allocation of weights determined by their relevance [7].

Another important step in the KDD process is data preprocessing [8], as the outcome of this stage is subject to exploration, and, therefore, it forms the basis for deriving the results of the data mining process. Proper data preparation and selection of relevant attributes can influence the algorithms used, their range, and operation, which, in turn, translates into the classification results and the discovery of patterns that exist in the input data.

Discretisation represents an aspect of data preprocessing. It involves the conversion of numerical attributes into discrete or categorical ones with a finite number of intervals [9]. It is classified as a data reduction method because it condenses a continuous spectrum of attribute values into a smaller set of discrete values. This simplification process helps to make the data more comprehensible, interpretable, and usable, while potentially eliminating noise. However, it can also result in some loss of information due to the disregard of some properties or relationships present in the continuous nature of attributes [10]. There are many discretisation methods and approaches, and the choice of a particular algorithm has an impact on the obtained discrete form of the features [11].

Supervised discretisation methods take into account class information to find proper intervals among the ranges of attribute values, as opposed to unsupervised algorithms, where the discretiser considers only the range of values being translated, and the number of intervals is given as an input parameter [12]. In the standard procedures used, transformations are applied to all continuous variables at once, before knowledge discovery, and the same mechanism is employed to translate all attribute domains [13].

Taking into account the above characteristics of the knowledge discovery and discretisation processes, in the framework of the research presented in this paper, the authors propose a methodology related to the data preprocessing stage, more specifically, data discretisation. The modification involves conditioning transformations of attribute domains on their relevance. The procedure of data discretisation is performed gradually, separately for each feature, and the selection of features is driven by constructed rankings [14]. As a consequence, not only the original continuous input space is explored, but all the partially discretised variants as well. The processing stops when all variables are translated into their discrete domains.

The proposed methodology was verified through extensive experiments, including two well-known methods for ranking construction, examined in both ascending and descending orders, for selected representatives of both supervised and unsupervised discretisation approaches, and three state-of-the-art classification algorithms. The procedure of gradual discretisation controlled by use of ranking was applied to the datasets from the stylometry domain with authorship attribution as a supervised machine learning task [15,16].

The research presented in the paper makes the following main contributions:

- Illustration of a research framework dedicated to a gradual discretisation procedure directed by selected rankings of features;
- Exploitation of multiple discretisation algorithms, with supervised and unsupervised interval construction;
- Comparison between domain transformations following rankings in ascending and descending directions;
- Analysis of trends in performance of state-of-the-art classifiers with varied operational backgrounds from the point of view of data representation and interpretation;
- Observation of the impact of considering information on the relevance of attributes during their discretisation on the performance of the selected classifiers;
- Application of the proposed methodology in the stylometric domain for authorship attribution tasks.

The paper is organised into six sections. Section 2 constitutes a presentation of the research background, with comments on important issues, methods, and tools employed. Section 3 provides a description of the framework of the research procedure in which attributes are discretised gradually, while following a ranking of attributes, with all relevant considerations. The range of experiments performed and their limitations and parameters are included in Section 4, while Section 5 is dedicated to analysis of the obtained results. Concluding remarks and possible future research directions are provided in Section 6.

## 2. Background

In this section, some aspects related to the nature of data are considered. In relation to the data preparation stage during the KDD process, approaches to data discretisation and methods of attributes ranking construction are described. Finally, popular classifiers used in the experiments are outlined.

### 2.1. Nature of Input Space

The nature of data refers to various aspects of the input space. It involves identifying characteristic features and their importance, including the type of data and their complexity, the structures in which they are stored, and the selection of tools, methods, and algorithms aimed at extracting knowledge from the data and discovering useful new patterns. Understanding the nature of data is crucial when designing systems for their processing, selecting analytical tools, and implementing appropriate data management and protection strategies. One of the important concerns in this context is the possible existence of data irregularities, which can be considered from various points of view [17]. This may involve issues related to data discretisation, the uneven distribution of decision classes within a dataset [18], or possible stratification visible in the data [19].

Knowledge mining algorithms often require data in a discrete form, necessitating the process of transformation for these data [20]. In cases where the data are continuous, their normalisation or standardisation could be needed to ensure that different attributes have a comparable scale. If discretisation is performed, it can lead to a loss of information regarding the relationships and dependencies present in the data. Transforming the data into a discrete form reduces memory usage and computational power requirements and ensures that the data are easier to understand and interpret [21]. However, selecting an appropriate data discretisation method is not a trivial task [22]. Standard approaches apply the transformations to all available features at once, and supervised methods are most often considered superior to unsupervised ones. In the investigations described, representatives of both approaches were involved, but discretisation was executed gradually on attributes, selected based on constructed rankings, which reflected how their importance was estimated.

The problem of imbalanced decision classes [23], where some classes are represented in the dataset to a significantly higher degree than others, can cause difficulties in classification, as algorithms may favour the dominant class. This, in turn, translates into a low accuracy of identification for objects belonging to minority classes and can lead to erroneous conclusions about the effectiveness of the model. Despite high overall accuracy, the model may struggle with accurately recognising important but less frequently occurring cases. In the conducted experiments, to ensure unbiased observations, the considered decision classes were balanced and had equal representation in all datasets.

Stratification, as a technique used in statistics and research, involves dividing a population or a dataset into smaller groups based on one or more attributes. The goal is to ensure the representativeness of the sample for the entire population, which allows for more accurate statistical estimations and translates into classification results. By reducing variance within groups, stratification can lead to a better understanding of population diversity and enables an analysis specific to individual groups. The application of this technique requires resources for data collection and comparative analysis with methods that overlook stratification. When stratification is a known characteristic of the input space, it needs to be taken into account in the performance evaluation step for algorithms involved in knowledge mining. In the performed experiments, stratification was applied and used during the division of datasets into the train and test sets for classification purposes.

All these characteristics of the input data significantly influence any KDD process. They affect every stage, from preprocessing to final analysis, and interpretation as well. Therefore, their understanding is critical for effective pattern recognition. They must be considered in the context of any observations or conclusions drawn from the executed tests.

### 2.2. Data Transformations

Within the realm of supervised machine learning, numerous algorithms rely on data in discrete forms. In this context, discretisation assumes a critical role during the stages of input data preparation. Its primary function involves converting numerical characteristic features into discrete or nominal ones with a finite number of intervals representing the attribute domains [24]. Discretisation serves as a means of reducing features, aiming to diminish the multitude of values associated with a continuous variable, by segmenting its range into bins. It can also provide some insight into how important the attributes are [25], which can lead to the reduction of entire domains by transforming them into a single categorical representation.

Typically, discretisation follows a series of steps [26]: (i) arranging the continuous values of an attribute to be discretised either in ascending or descending order; (ii) determining and assessing cut-points to divide a range of continuous values or combine neighbouring intervals; (iii) dividing or merging intervals of the attribute’s values based on the chosen discretisation method and criteria; (iv) verifying the stopping criterion using a measure to regulate the entire discretisation process.

Discretisation techniques can be categorised based on various criteria. Among the most commonly recognised classifications are supervised versus unsupervised, local versus global, static versus dynamic, and top-down versus bottom-up approaches [9]. The properties associated with any specific processing are reflected in how intervals are constructed and the cut-points between them selected, and how many categorical values are defined for the variables.

In contrast to supervised methods, unsupervised algorithms disregard instance labels when transforming attribute values [11]. Local approaches focus on a subset of the discretised object space, while global methods consider the entire instance space. Dynamic discretisation involves examining interdependencies among variables, while static methods treat each attribute independently. In top-down processing, a large range is divided into smaller intervals, whereas in the bottom-up approach, small original intervals are merged into larger ones.

Discretisation algorithms from the group of supervised methods are widely considered as resulting in obtaining the most advantageous representation of the data in a discrete domain. Popular standard approaches from this category are Fayyad and Irani [27] and Kononenko [28]. They rely on the class entropy [29] within the intervals under consideration to evaluate cut-points and utilise the Minimum Description Length (MDL) principle [30,31] as a stopping criterion. The process of determining cut-points works in a top-down direction. It begins with a single interval that encompasses all values of the attribute to be discretised. Partitioning continues recursively until a stopping criterion is satisfied.

For the Fayyad and Irani method, firstly, the class entropy Ent(S) is calculated:

(1) $$Ent\left(S\right)=-\sum_{i=1}^{k}P\left(C_{i},S\right)\log\left(P\left(C_{i},S\right)\right),$$

where *S* is a set of *N* instances with *k* decision classes $C_{1},\ldots ,C_{k}$, and $P(C_{i},S)$ is the proportion of class $C_{i}$ instances in *S*.

For the case of binary discretisation of a continuous attribute *A*, the optimal selection of cut-point $T_{opt}$ is performed by testing and evaluating all possible candidate cut-points *T*. The entropy for a cut-point *T*, which splits the set *S* into two subsets, $S_{1}$ and $S_{2}$, where the $S_{1}\subset S$ contains instances with attribute values ≤T and $S_{2}=S\setminus S_{1}$, is calculated as follows:

(2) $$Ent\left(A,T;S\right)=\frac{|S_{1}|}{\left|S\right|}Ent\left(S_{1}\right)+\frac{|S_{2}|}{\left|S\right|}Ent\left(S_{2}\right).$$

For the optimal cut-point $T_{opt}$, the class information entropy $Ent(A,T_{opt};S)$ is minimal.

The stopping criterion referring to the MDL principle is connected with the information gain:

(3) Gain(A,T;S)=Ent(S)−Ent(A,T;S).

The discretisation process is applied recursively until the inequality (4) is satisfied,

(4) $$Gain\left(A,T;S\right)>\frac{\log_{2}\left(N-1\right)}{N}+\frac{\Delta (A,T;S)}{N},$$

where

(5) $$\Delta \left(A,T;S\right)=\log_{2}\left(3^{k}-2\right)-\left[k\cdot Ent\left(S\right)-k_{1}\cdot Ent\left(S_{1}\right)-k_{2}\cdot Ent\left(S_{2}\right)\right].$$

In the case of the Kononenko method, the discretisation process is applied recursively until the inequality (6) is satisfied,

(6) logNNC1…NCk+logN+k−1k−1>∑jlogNAjNC1Aj…NCkAj+∑jNAj+k−1k−1+logNT,

where

*N*—the number of training instances,
$N_{C_{i}}$—the number of training instances from the class $C_{i}$,
$N_{A_{x}}$—the number of instances with the *x*-th value of the given attribute,
$N_{C_{i}A_{y}}$—the number of instances from class $C_{i}$ with the *y*-th value of the given attribute,
$N_{T}$—the number of possible cut-points. 

The two most commonly used representatives of the unsupervised approaches are equal-width binning and equal-frequency binning. For both, the number of intervals *k* to be constructed is defined by a user [9]. For the two algorithms, the values of a continuous attribute are sorted and the minimum and maximum values of the discretised feature are identified. In the case of the equal-width method, the range of attribute values is divided into *k* equal-width discrete intervals. In the case of equal-frequency binning, the range is divided into *k* intervals such that each bin contains the same number of sorted values.

Both techniques are relatively straightforward but can be influenced by the number of bins specified by the user. A drawback is that when values of a continuous attribute are unevenly distributed, the discretisation process may result in the loss of some information [22]. In the case of the equal-frequency method, numerous instances of a continuous value might lead to that value being allocated into different bins. Therefore, during the determination of cut-points, it is crucial to ensure that duplicate values are assigned to one bin only. In the case of the equal-width algorithm, intervals can be defined for regions of space where no datapoints exist.

In standard discretisation approaches, all attributes receive the same treatment and are processed at the same time, typically in the data preprocessing step, before data exploration. In the paper, a different way of proceeding is illustrated through the proposed methodology, where discretisation is performed gradually, with transformation of one attribute at a time. In the investigations carried out, selected representatives of both supervised and unsupervised discretisation methods were used, and the procedure involved taking into account the importance of the features to be discretised.

### 2.3. Importance of Attributes

Feature selection can be executed through two distinct methods: by choosing a subset of attributes or by establishing a ranking of variables based on their significance [32,33]. In both cases, the main goal is to reduce the dimensionality of the data by eliminating irrelevant or redundant attributes. It helps in building more efficient and faster models and facilitates the interpretation of the data. Decreasing the number of features can also reduce the computational and memory requirements.

Creating a ranking of attributes involves evaluating and ordering the features available in a dataset according to their significance or impact on a specific analytical objective, such as model prediction. Various methods can be utilised for ranking construction [34]: statistical tests, entropy-based methods, principal component analysis, or machine learning algorithms, e.g., random forests offer built-in feature importance assessment mechanisms [35]. Some approaches apply a scoring function whose values can be treated as assigned weights, while others, for example, sequential search [36], just return an ordering of attributes. The evaluation of features allows for the identification of the most significant ones and their ordering from the most to the least important, or in reverse order.

Relief and OneR are popular ranking mechanisms, which were studied in the research framework presented. Their implementation is available in the WEKA software [37]. They belong to the category of algorithms that treat all available attributes as relevant and always assign a non-zero score. Both algorithms can handle categorical as well as numerical types of features.

The Relief algorithm falls under methods that rely on the instances present in the training data [38]. When it is applied, each variable accumulates a score that indicates its effectiveness in distinguishing between different classes. At the beginning of the algorithm, all the features are assigned weights with a value of zero. In the iterative process, the nearest instance (neighbour) of the same class (nearest hit *H*) and the nearest instance of a different class (nearest miss *M*) are identified. Based on the calculated differences between the feature values of the current instance and its nearest hit and nearest miss instances, the weights of the attributes are updated. Higher scores are assigned to those attributes that demonstrate larger differences for nearest hits and smaller differences for nearest misses. The pseudo-code is listed as Algorithm 1.
**Algorithm 1** Pseudo-code for Relief**Input**:    set of learning instances *X*,            set *A* of all *N* attributes,            set of classes ***Cl***,            probabilities of classes *P*(*Cl*),            number of iterations *m*,            number *k* of considered nearest instances from each class; **Output**: vector of weights w for all attributes; **begin** **for** i = 1 **to**
*N*
**do**            *w*(*i*) = 0 **end for** **for** i = 1 **to**
*m*
**do**            choose randomly an instance *x* ∈ *X*            find *k* nearest hits *H_j_*            **for** each class *Cl* ≠ *class*(*x*) **do**              find *k* nearest misses *M_j_*(*Cl*)            **end for**            **for**
*l* = 1 **to**
*N*
**do**              $w(l)=w(l)-\sum_{j=1}^{k}\frac{\mathrm{diff}(l,x,H_{j})}{m\times k}+\sum_{\mathrm{Cl}\neq \mathrm{class}(x)}\frac{\frac{P(\mathrm{Cl})}{1-P(\mathrm{class}(x))}\sum_{j=1}^{k}\mathrm{diff}(l,x,M_{j}(\mathrm{Cl}))}{m\times k}$            **end for** **end for** **end** {algorithm}


The difference function for categorical attributes returns one if the values are distinct, and zero if they are the same. For numerical attributes, it provides the normalised difference. After iterating through the dataset, the weights assigned to the features represent their importance. Attributes with higher weights are considered more relevant for classification as they contribute more effectively to discrimination between classes [39].

OneR (One Rule) is a simple and effective algorithm used to evaluate the importance of features in a dataset during classification tasks [40]. It examines attributes individually and ranks them based on their ability to discriminate between different classes in the dataset. For each unique value of the chosen feature, the algorithm generates one rule, collectively forming the basis of the classification model. The feature selected by OneR is the one that results in the lowest error rate when predicting the class labels. This algorithm is a straightforward approach to feature ranking; it can provide valuable insight regarding which features are the most informative for classification [41]. However, it may not always capture complex relationships between features, and its effectiveness can vary depending on the dataset and the nature of the problem. Algorithm 2 presents the pseudo-code of the OneR algorithm.
**Algorithm 2** Pseudo-code for OneR classifier**Input**:    set *A* of all attributes,            set of learning instances *X*; **Output**: 1-rule 1-*r_B_*; **begin** *CandidateRules*←Ø **for** each attribute *a* ∈ *A*
**do**            **for** each value *v_a_* of attribute *a*
**do**              count how often each class appears              find the most frequent class *Cl_F_*              construct a rule *IF*
*a* = *v_a_*
*THEN*
*Cl_F_*            **end for**            calculate classification accuracy for all rules            choose the best rule *r_B_*            *CandidateRules*←*r_B_* **end for** choose as 1-*r_B_* the best one from *CandidateRules* **end** {algorithm}


### 2.4. Exploration of Input Space

Classification stands as a fundamental activity within the realms of knowledge discovery and pattern recognition. It can be viewed as a function that assigns a class label to the instances characterised by a set of attributes. In this work, three state-of-the-art classifiers were employed, including Naive Bayes, J48 and *k*-Nearest Neighbours.

The Naive Bayes (NB) classifier is a probabilistic machine learning algorithm used for classification tasks. It is based on Bayes’ theorem, which describes the probability of an event, based on prior knowledge of conditions that might be related to the event [42]. The “naive” part of its name comes from the assumption of independence among features. NB assumes that the presence of a particular feature in a class is unrelated to the presence of any other feature. This simplifies the computation and makes it more efficient, although it is often an oversimplification of real-world scenarios. Despite their simplicity and the “naive” assumption, Naive Bayes classifiers often perform well, especially with high-dimensional data, and they are computationally efficient, making them a popular choice for many classification problems [43].

J48 is a decision tree algorithm used in the field of machine learning and data mining. It is an implementation of the C4.5 algorithm, created by R. Quinlan [44]. J48 is often used in data classification and prediction. The name “J48” refers to the file format that creates the decision tree. The algorithm constructs a data model in the form of a decision tree. In the structure of this tree, every internal node represents an attribute, and each terminal node, referred to as a leaf, corresponds to a class label. By traversing the tree from the root to the leaves, a decision can be made for a given object under consideration. This approach allows understanding the rationale behind a specific decision and offers a straightforward and intuitive way to represent complex decision-making processes. Thus, decision trees are not only recognised as effective classifiers but also serve as a widely adopted form of knowledge representation [45].

The *k*-Nearest Neighbours (k-NN) classifier is a simple and intuitive machine learning algorithm that operates on continuous as well as discrete data [46]. The parameter *k* represents the number of neighbours to consider. They are identified on the basis of a distance metric that depends on the nature of the data—very often the Euclidean distance is used. In the framework of a classification task, the k-NN algorithm classifies the given new object by finding the majority class among its *k* nearest neighbours in the feature space. In regression tasks, instead of class labels, the algorithm predicts a continuous value by averaging the values of the *k* nearest neighbours. k-NN is a non-parametric algorithm which is categorised as a lazy learning method—it does not make any assumptions about the underlying data distribution and it does not learn a model during the training phase. Instead, it stores all the training data and makes predictions only when required during the testing phase. The latter property means that in the case of a large data size, the computational cost of determining the distance between objects increases, which significantly affects the performance of the algorithm.

## 3. Framework for Discretisation Controlled by Attribute Importance

The current investigation sought to provide as unbiased observations on the experiments as possible. Therefore, the algorithm for selective discretisation of the characteristic features, controlled and directed by their rankings, required certain limitations, assumptions, and decisions on the processing paths to take. This section provides comments on all the relevant considerations and explanations for all the steps.

### 3.1. Input Data and Attributes

All attributes are expected to be of the same fundamental nature, with the continuous domains and comparable ranges of their values. Only then are their representations before and after discretisation transformations similar. Variables should be selected based only on domain knowledge, without applying algorithms dedicated to feature selection, otherwise such double processing would make distinguishing their individual impacts impossible. To avoid the influence of imbalance or the existence of multiple classes on recognition, the classification task to be solved is binary, with balanced classes, and where both classes are considered to be of the same importance.

### 3.2. Rankings

Some ranking mechanisms evaluate and then select a subset of attributes, while to the remaining ones a zero rank is assigned. In effect, this means rejecting such non-ranking variables as entirely irrelevant. This could result from applying the notion of entropy in the evaluation of features, which could lead to finding that they do not support class recognition. The methods to be used in the proposed framework must belong to the other category of weighting approaches, which treat all the variables as relevant to some non-zero degree, by always assigning a rank different from zero. It is not necessary for any specific score to be given to the features, as the focus is only on the ordering obtained. The evaluation of importance must take place in the continuous domain. A ranking is assumed to involve a standard ordering of attributes, with the most important features at the top, and the least important variables placed at the bottom.

### 3.3. Discretisation Approaches

A static discretisation process is required, where transformations are executed independently on a learner used. All attributes should be separately translated into a discrete domain, and the algorithm should be univariate, that is, not taking into account any interdependencies among the variables. For transformations, supervised as well as unsupervised methods can be used. However, typically, supervised discretisation reflects to some extent how variables support recognition, which can be perceived as their importance, making it similar to ranking procedures. Furthermore, in a top-down algorithm, which starts with constructing a single interval to represent the entire range of continuous values translated to a discrete domain, if all the candidate cut-points are evaluated and rejected, then this single bin remains a sole discrete value for an attribute. In such a case, the attribute is practically removed from considerations in a discrete domain, as nothing can be learnt from its constant value in all samples.

### 3.4. Inducers

To observe the impact of the discretisation of attributes on the performance of a classifier, the inducer is required to be capable of efficient operation on both continuous and nominal values of variables, without any inherent transformations of features corresponding to discretisation. Since learners are sensitive to forms of attributes in varying degrees, more varied mathematical backgrounds and diverse modes of operation of employed classification systems provide a wider scope of observations.

### 3.5. Starting and Stopping Point

The processing starts with exploration of the datasets in the continuous domain. The performance is evaluated by labelling previously unknown samples in the test sets with reference to knowledge discovered in the train sets, expressed through patterns detected in the real-valued variables. The performance observed in this step constitutes one of the reference points for comparisons in further processing.

The stopping point for the procedure is reached once the set of variables is exhausted, when all are processed, and the entire datasets become discrete. The performance of inducers for the discretised data is the second reference point. Transformations can also be stopped sooner, when some noticeable worsening or increase in performance is observed. However, it can result in missing global maxima and too narrow a focus on some local trends in monotonicity.

### 3.6. Intermediate Steps and Directions of Processing

The pseudo-code for the ranking-driven discretisation procedure is shown in Algorithm 3. At each processing step, a single attribute is discretised. The variables chosen for the transformation are indicated by their position in a ranking. The ranking can be followed either in descending order, starting with the top positions taken by the most important features and then of gradually decreasing relevance, or in ascending order, beginning with the least important variables, placed at the bottom of the ranking, and then climbing up the ranks. Therefore, putting aside the starting point (all attributes continuous) and the stopping point (all variables discretised), for the rest of the processing, the datasets are partially continuous and partially discrete, and the number of middle steps to take equals the number of available features minus one.
**Algorithm 3** Pseudo-code for ranking driven discretisation**Input**:    ranking of attributes *Ranking_A_*,            dataset in the continuous domain *Data-R*,            direction *Direction* to pursue ranking *Ranking_A_*,            number of attributes *N*; **begin** *TMP-Data*←*Data-R* mine knowledge from *TMP-Data* evaluate performance for *TMP-Data* **if** *Direction* = Descending **then**
*k* = 1 **else**
*k* = *N* **while** (*k* > 0) AND (*k* < *N* + 1) **do**            select attribute from the ranking *attr* = *Ranking_A_*[*k*]            discretise *attr* in *TMP-Data*            mine knowledge from *TMP-Data*            evaluate performance for *TMP-Data*            **if**
*Direction* = Descending **then**
*k* = *k* + 1 **else**
*k* = *k* − 1 **end while** **end** {algorithm}


## 4. Experiments

The experiments that were carried out began with preparation of the input datasets, with all attributes in the continuous domain. For the available features, the rankings were calculated next. Then, the procedure of selective discretisation controlled by a ranking was applied to the data. All data variants, continuous, partially discrete, and completely discrete, were explored by the selected classifiers. Performance was studied in the context of data form, ranking, and inducer.

The research included L=2 rankings, examined in both ascending and descending order. They were applied to N= 12 attributes, and exploited in the gradual discretisation procedure with M=20 discretisation approaches tested (two supervised discretisation methods, and two unsupervised discretisation methods with nine variants each). Therefore, per dataset, $1+M\left(2L(N-1)+1\right)$ versions of the data (making a total of 901) were explored by three selected classifiers, and their performance was evaluated with the test sets, discretised accordingly. The parameters of these extensive experiments are commented on in this section, while the results obtained are shown and discussed in the next one.

### 4.1. Data Preparation

To minimise the number of factors that could influence and bias the results of the experiments, a binary authorship attribution was selected as a classification task under study. The problem of attribution of authorship belongs to the stylometric domain [47]. It can be treated as a classification task by training an inducer on samples of known authorship to detect the linguistic characteristics of writing styles. It leads to the construction of stylistic profiles for authors [48]. Then, such profiles are matched to text samples of questionable origin to either confirm authorship, or to deny it [49].

For the stylometric analysis, two pairs of well-known writers were taken: the literary works of Edith Wharton and Mary Johnston formed the basis for the female writer dataset (F-writers), and the novels by Henry James and Thomas Hardy were used for the construction of the male writer dataset (M-writers). To increase the numbers of available text samples, these long texts were partitioned into much smaller parts, keeping comparable lengths [50]. For all these text chunks, the values were calculated for the arbitrarily selected group of lexical descriptors [51] in the form of frequency of occurrence [52] for twelve common two-letter function words as follows: as, at, by, if, in, no, of, on, or, so, to, up. Since these attributes are regular words, when they are referred to in descriptions of the experiments, formatting in italics is employed (e.g., the frequency of occurrence of *of*).

Due to the division of the novels into smaller parts, the samples obtained were grouped in the input space by these longer works, which led to a stratified space. In such a situation, to arrive at reliable observations, the original works need to be separated into sets to be used for training and testing. Samples based on one and the same novel are more similar, and using them for both stages would result in leaking information and overoptimistic predictions. The conditions for such unreliable evaluation would be given by the popular standard cross-validation technique employed in estimations of performance [19].

Cross-validation relies on multiple execution of train and test procedures, over which an average is calculated, and samples for all steps are selected randomly. In the stratified space, where groupings of points form a specific and known pattern, a completely random selection would increase the probability of biased recognition. To avoid this problem, non-standard cross-validation can be used, not exchanging single samples but rather their groups between the training and test sets. However, such processing results in very high additional computing costs. As a compromise, a different approach can be employed, with a single train and multiple test sets, all constructed based on separate long texts. Then, with respect to performance, the predictions averaged over all test sets are reported. The latter approach was implemented in the research. Apart from the train set, each dataset included two test sets. All the original sets were continuous with balanced data.

### 4.2. Rankings Employed

In the investigations, two ranking mechanisms were applied to the available features with the continuous domains. Relief and OneR are popular algorithms, implemented in the WEKA workbench [37]. Both rankings assign a non-zero rank to all the available attributes, treating all of them as relevant to some extent. For the two rankings, the resulting order of attributes for the male and female writer datasets is shown in Table 1.

Although the studied datasets share stylometric features, their role for each dataset is considered locally; therefore, they were mostly placed differently in the rankings. For the F-writers the orderings obtained by Relief and OneR were relatively close, in particular in the upper half, for the more important attributes. For the M-writers, there were more differences noted. Despite the similarities, the two rankings were not identical, and both were exploited in the next stage of the experiments as indicators of attributes to discretise one-by-one in a sequential process.

When a ranking was processed upward (in an ascending direction), it meant starting at the bottom of the ranking, with the least important variables and transforming them before moving on to the more relevant variables. Going down the ranking (in a descending direction) was understood as translating in the first discretisation steps the most important features, with the highest ranks, placed at the top of the ranking, and only then proceeding to less relevant variables.

### 4.3. Discretisation Algorithms

The discretisation methods used were from both supervised and unsupervised categories [24]. Unsupervised equal-width binning (duw) was employed, varying the number of bins to be constructed from two to ten, which returned nine variants of the datasets (duw02 ÷ duw10). In the same way, nine discrete variants of the data were obtained by applying unsupervised equal-frequency binning (duf), with the number of intervals ranging from two to ten (duf02 ÷ duf10).

The supervised discretisation methods (ds), that is, the Fayyad and Irani (dsF) and Kononenko (dsK) approaches, returned single variants of the data. These two algorithms rely on the MDL principle and calculation of entropy when the construction of intervals is performed. It led to some variables for which one interval was found to represent the entire range of values in a discrete domain. These attributes were effectively excluded from the data mining that followed discretisation. For F-writers, for both the supervised discretisation algorithms, there were six such features (*if*, *in*, *no*, *or*, *so*, *up*). For M-writers and dsK, six variables also had only single bins (*as*, *no*, *on*, *so*, *to*, *up*). For dsF, this set was expanded to seven elements (by adding *of*).

When a dataset consists of some constituent sets with the same input features, several different approaches to their discretisation can be attempted [53], all with some advantages and disadvantages, in particular, when irregularities in the data are observed [17]. In the experiments, all the sets were discretised independently, based on the characteristics of each individual set.

### 4.4. Performance Evaluation for Classifiers

In the investigations, three types of classification systems were used, Naive Bayes, J48 and k-NN. The differences in their operation mode and mathematical foundations enabled widening the scope for observations. As the goal of the research was to discover the relations between the attributes’ importance and their form, either continuous or discrete, and how they reflect on the performance of inducers, some measure for the estimation of quality was needed. Classification accuracy was chosen as the suitable indicator, showing how good the inducers were overall at attributing the considered authors to the text samples.

The samples to be labelled were entirely unknown to the classifiers and came from long texts that were not used for training. This way of proceeding prevented possible bias in recognition. The samples were grouped into two test sets; predictions obtained for them were averaged, and then, finally, reported as the percentage of texts for which correct authors were found in relation to the total number of samples.

For all inducers, their powers were studied under three conditions. The starting point was in a continuous domain, with all variables real valued. The end point was in a discrete domain, with all features discretised, for all variants of discrete datasets. And, in addition, a space was studied where some features were still continuous while others were discretised.

## 5. Results and Their Discussion

The results of the experiments can be studied from several perspectives. The performance of classifiers in the original input space needs to be contrasted with the results obtained for all variants of discretised space, with partial or complete transformations of the attributes. The first part of this section includes comments regarding reference points; the second part shows the performance trends observed inside the procedure of gradual discretisation. The third part is dedicated to an examination of the ranges of classification accuracy obtained, using selected calculated statistics.

### 5.1. Reference Points

To find out if the overall change in representation for attributes from continuous to discrete was advantageous to recognition by a classifier, the performance at the starting point, with all variables real-valued, needs to be compared with the performance at the finish line, with all attributes discretised, which then both constitute reference points. For all three inducers used in the described research, the classification accuracy, evaluated by labelling samples from the test sets, is shown in Table 2. Each column of the table shows the performance of a specific inducer for a dataset, with each row listing the result obtained for a certain data variant, either continuous or discrete. The highest accuracy discovered is marked in bold.

From these results, it can be observed that for all three inducers and both datasets, translation from the continuous into a discrete domain was not always advantageous. However, only for the Naive Bayes classifier, for M-writers, the maximum was obtained in the original input space before transformations. For all variants of the discretisation procedures, when translation was executed for all features, predictions in this case were brought down. On the other hand, for the NB and the female writer dataset, maximum precision was found for unsupervised discretisation with the equal-frequency binning approach, with seven bins constructed for all variables.

For the other two classifiers, J48 and k-NN, the maximum classification accuracy was detected in discrete spaces. For the J48 and the F-writers, it was for supervised discretisation by the Kononenko algorithm, and for M-writers, again for duf binning with 10 intervals defined. On the other hand, for k-NN, the maximum was found for M-writers for the Fayyad and Irani discretisation processing, and for F-writers, once again for unsupervised discretisation by equal-frequency binning, for six bins.

Since supervised discretisation is popularly considered superior to unsupervised transformations, it is worth observing that this opinion was not confirmed in these experiments. There were cases where supervised processing led to better results, but also conditions occurred when unsupervised algorithms returned variants of the data that caused improved predictions. However, a maximum was never found for processing with unsupervised equal-width binning, regardless of the number of intervals defined.

### 5.2. Performance Trends

**Figure 1:**
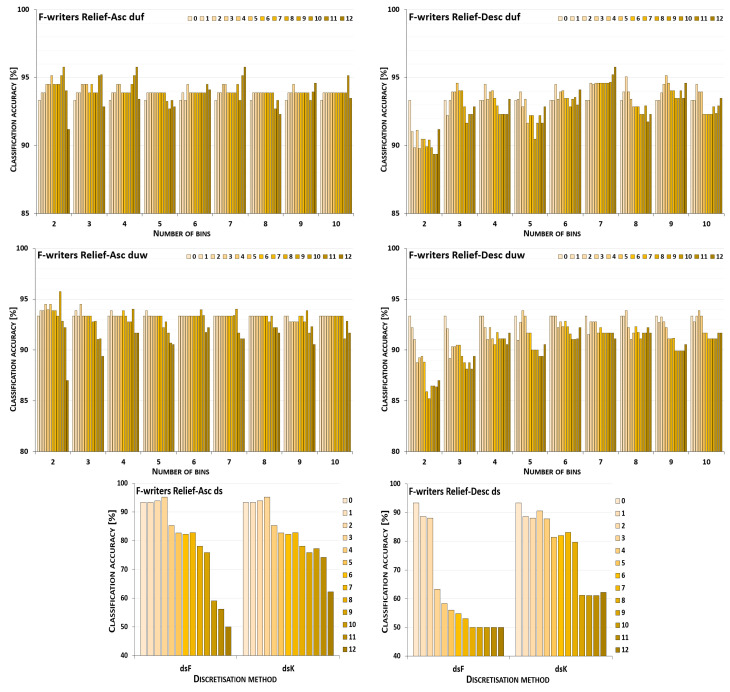
Performance [%] for the Naive Bayes classifier observed in the discretisation of the female writer dataset while following the Relief ranking. For unsupervised equal-frequency (duf) and equal-width (duw) binning, the categories reflect the number of constructed bins, and for supervised approaches, the method is given. The series specify the number of discretised attributes.

**Figure 2:**
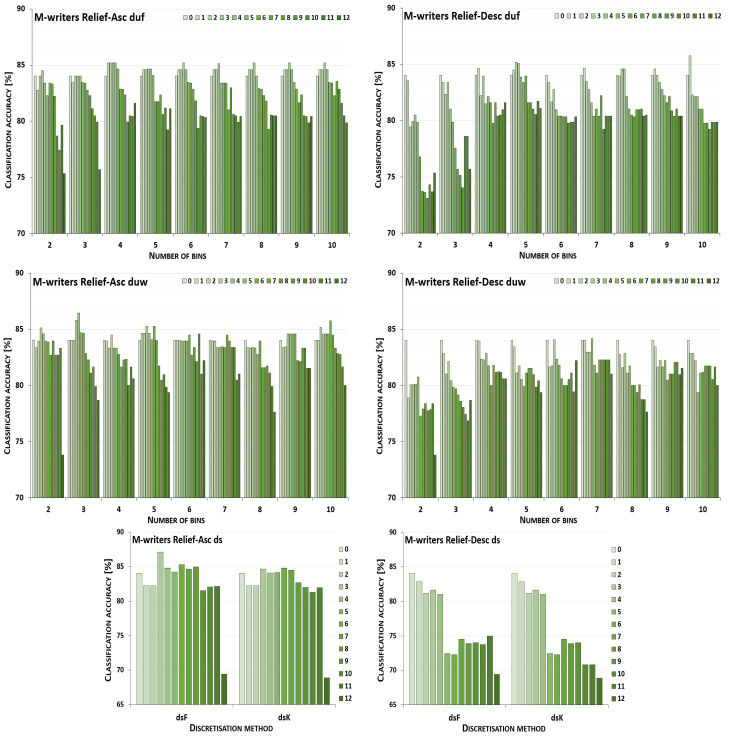
Performance [%] for the Naive Bayes classifier observed in the discretisation of the male writer dataset while following the Relief ranking. For unsupervised equal-frequency (duf) and equal-width (duw) binning, the categories reflect the number of constructed bins, and for supervised approaches, the method is given. The series specify the number of discretised attributes.

**Figure 3:**
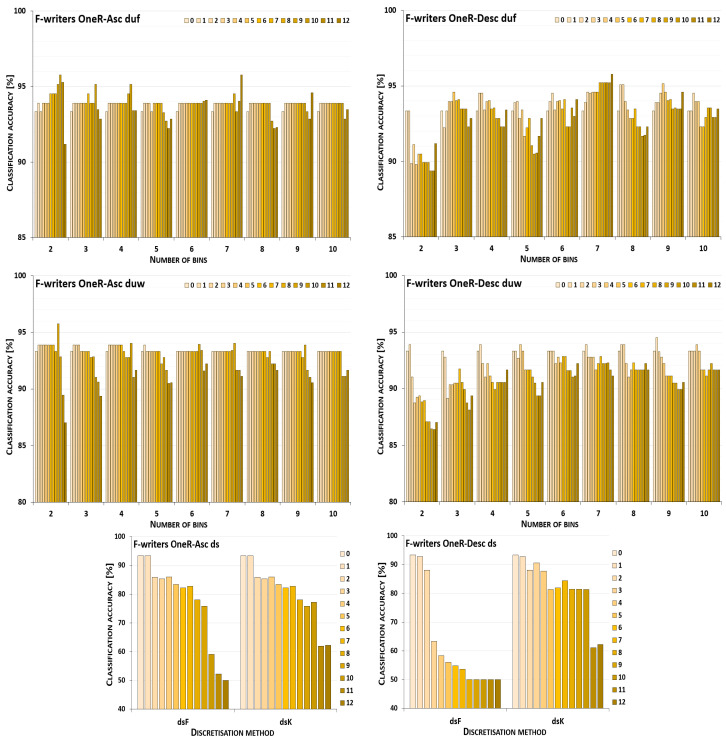
Performance [%] for the Naive Bayes classifier observed in the discretisation of the female writer dataset while following the OneR ranking. For unsupervised equal-frequency (duf) and equal-width (duw) binning, the categories reflect the number of constructed bins, and for supervised approaches, the method is given. The series specify the number of discretised attributes.

**Figure 4:**
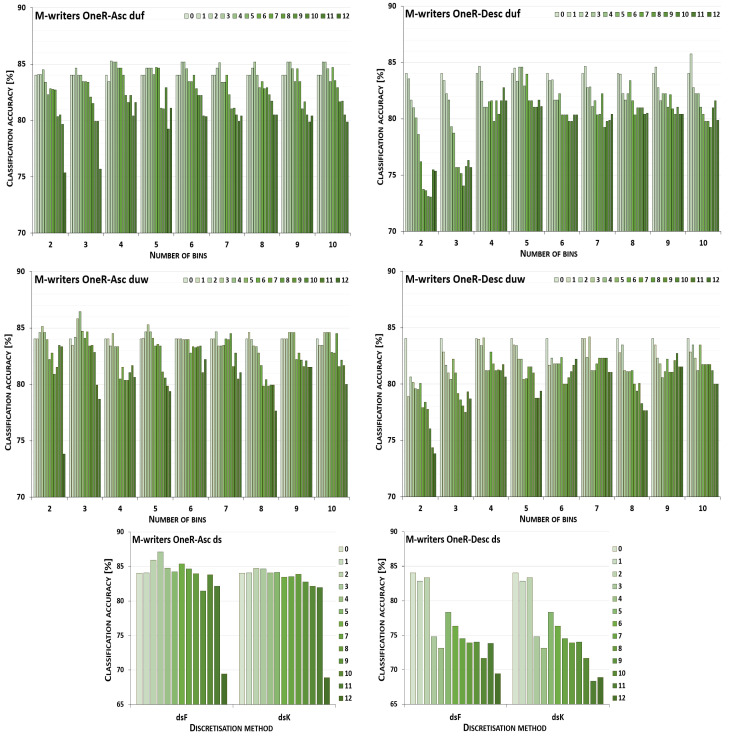
Performance [%] for the Naive Bayes classifier observed in the discretisation of the male writer dataset while following the OneR ranking. For unsupervised equal-frequency (duf) and equal-width (duw) binning, the categories reflect the number of constructed bins, and for supervised approaches, the method is given. The series specify the number of discretised attributes.

**Figure 5:**
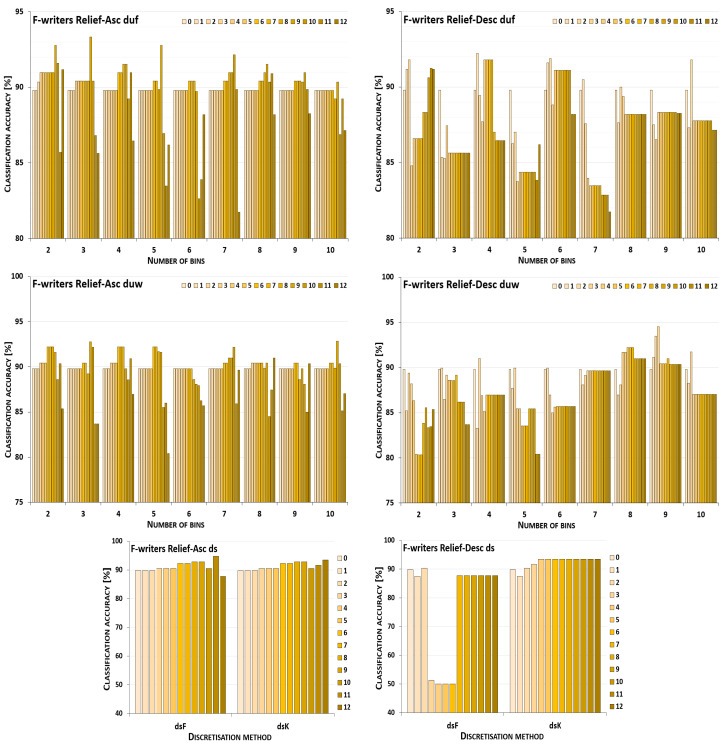
Performance [%] for the J48 classifier observed in the discretisation of the female writer dataset while following the Relief ranking. For unsupervised equal-frequency (duf) and equal-width (duw) binning, the categories reflect the number of constructed bins, and for supervised approaches, the method is given. The series specify the number of discretised attributes.

**Figure 6:**
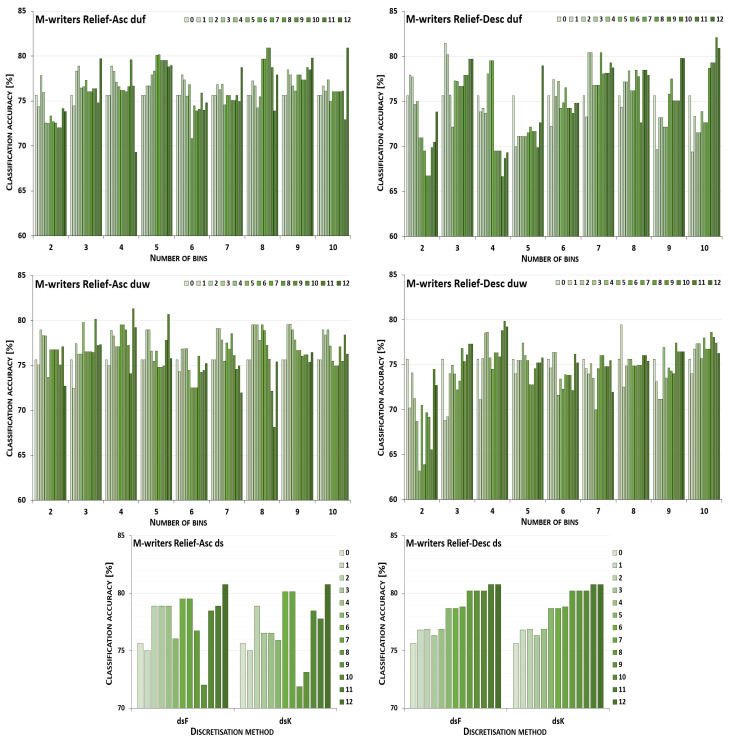
Performance [%] for the J48 classifier observed in the discretisation of the male writer dataset while following the Relief ranking. For unsupervised equal-frequency (duf) and equal-width (duw) binning, the categories reflect the number of constructed bins, and for supervised approaches, the method is given. The series specify the number of discretised attributes.

**Figure 7:**
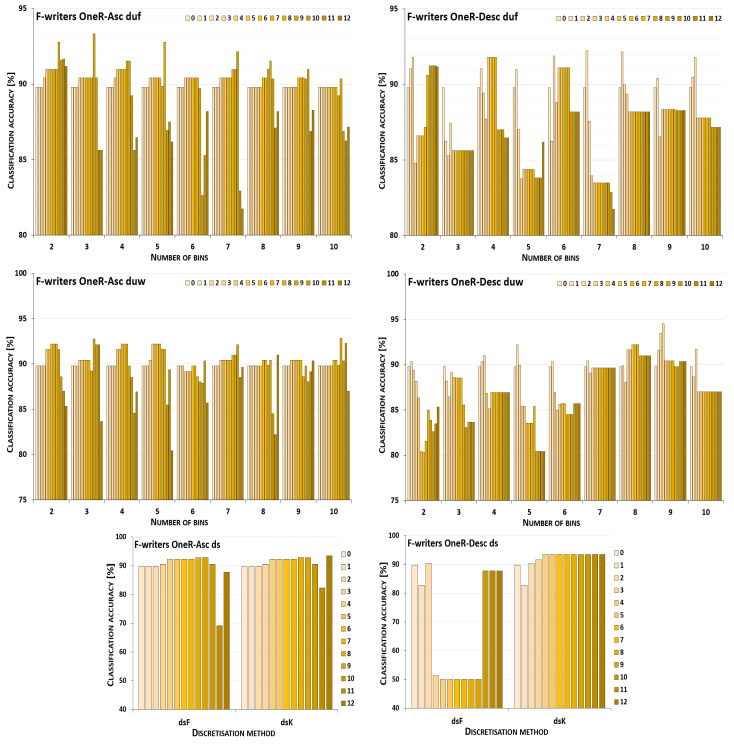
Performance [%] for the J48 classifier observed in the discretisation of the female writer dataset while following the OneR ranking. For unsupervised equal-frequency (duf) and equal-width (duw) binning, the categories reflect the number of constructed bins, and for supervised approaches, the method is given. The series specify the number of discretised attributes.

**Figure 8:**
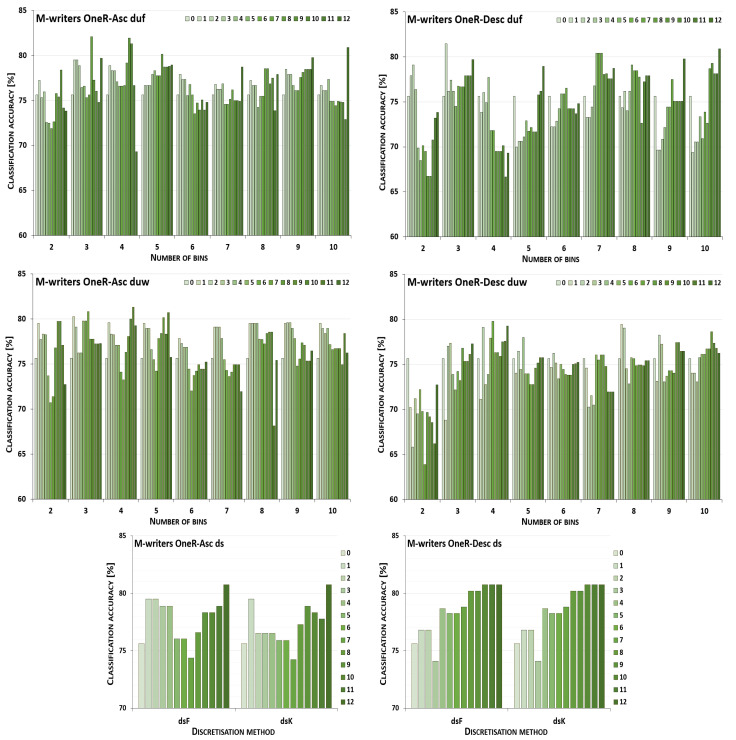
Performance [%] for the J48 classifier observed in the discretisation of the male writer dataset while following the OneR ranking. For unsupervised equal-frequency (duf) and equal-width (duw) binning, the categories reflect the number of constructed bins, and for supervised approaches, the method is given. The series specify the number of discretised attributes.

**Figure 9:**
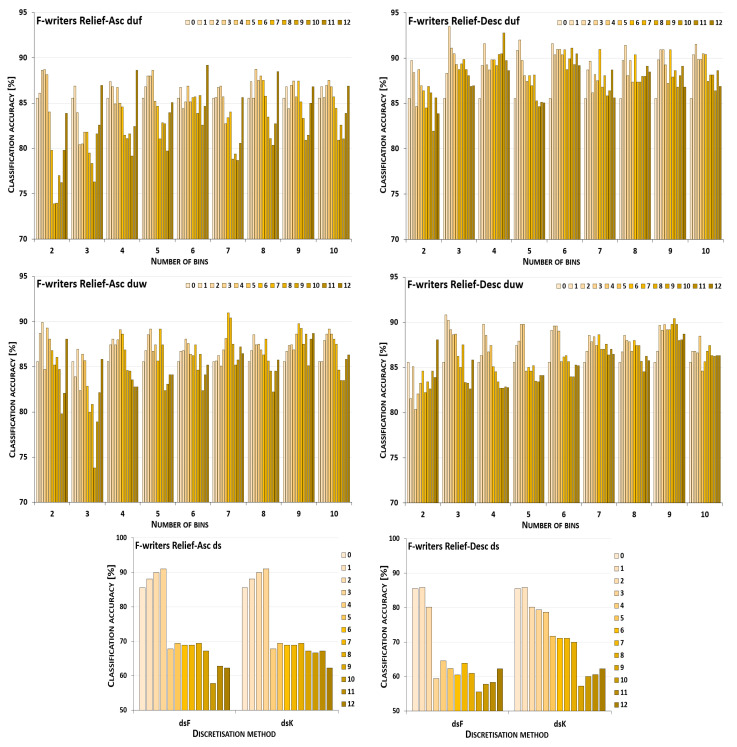
Performance [%] for the k-NN classifier observed in the discretisation of the female writer dataset while following the Relief ranking. For unsupervised equal-frequency (duf) and equal-width (duw) binning, the categories reflect the number of constructed bins, and for supervised approaches, the method is given. The series specify the number of discretised attributes.

**Figure 10:**
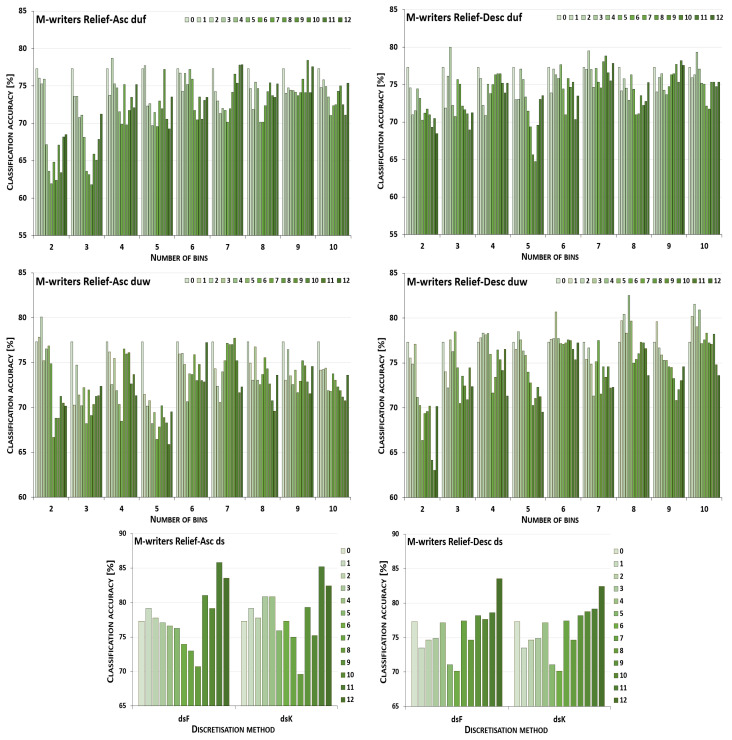
Performance [%] for the k-NN classifier observed in the discretisation of the male writer dataset while following the Relief ranking. For unsupervised equal-frequency (duf) and equal-width (duw) binning, the categories reflect the number of constructed bins, and for supervised approaches, the method is given. The series specify the number of discretised attributes.

**Figure 11:**
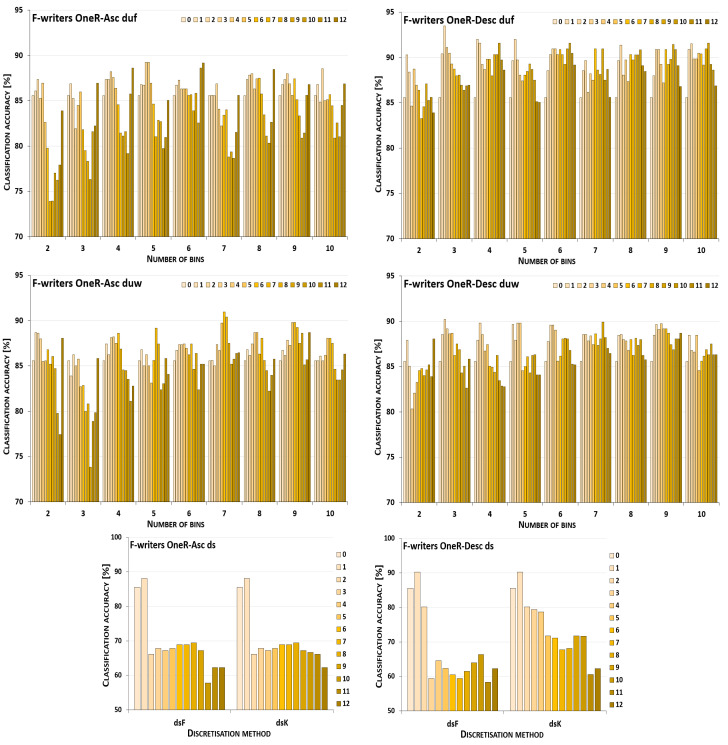
Performance [%] for the k-NN classifier observed in the discretisation of the female writer dataset while following the OneR ranking. For unsupervised equal-frequency (duf) and equal-width (duw) binning, the categories reflect the number of constructed bins, and for supervised approaches, the method is given. The series specify the number of discretised attributes.

**Figure 12:**
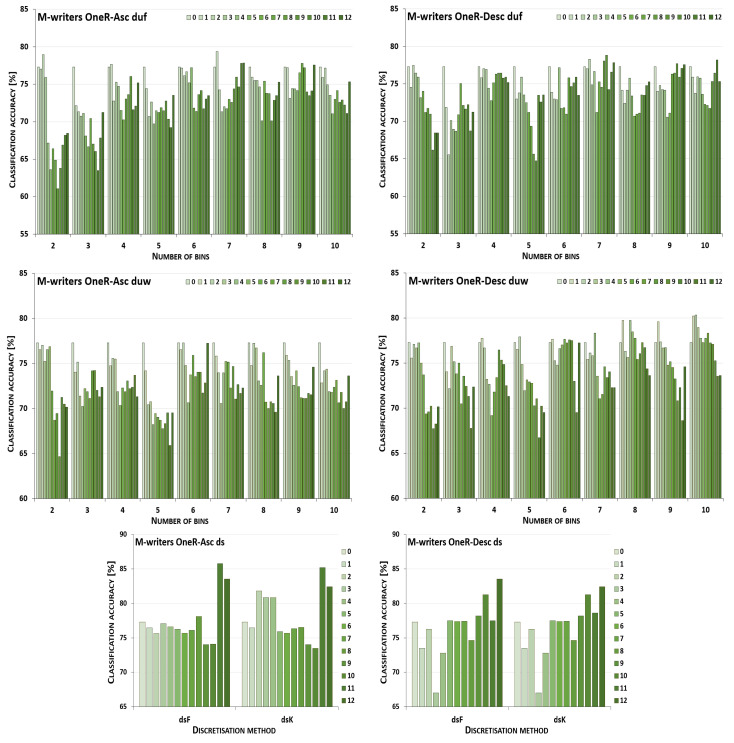
Performance [%] for the k-NN classifier observed in the discretisation of the male writer dataset while following the OneR ranking. For unsupervised equal-frequency (duf) and equal-width (duw) binning, the categories reflect the number of constructed bins, and for supervised approaches, the method is given. The series specify the number of discretised attributes.

The discretisation process referring to the importance of attributes shown by a ranking was studied for the three inducers in the context of a particular discretisation method, ranking, and direction of transformations. The results obtained are displayed in Figure 1, Figure 2, Figure 3, Figure 4, Figure 5, Figure 6, Figure 7, Figure 8, Figure 9, Figure 10, Figure 11 and Figure 12. Each figure shows the performance reported by a classifier for one ranking, for both ascending (left half of a figure) and descending (right part of a figure) directions, for all four types of discretisation algorithms, for either the female or male writer dataset.

In the charts showing the performance of classifiers, for the unsupervised discretisation methods, the categories on the x-axis show the number of bins constructed for the transformed variables, and for supervised discretisation, the method is given. The data series specify the number of attributes after discretisation, where 0 means that all variables were continuous (the original input space before any transformations), and 12 denotes the situation where the entire set of available features was translated (the final form, with all discrete attributes). The charts on the left display processing in an ascending direction of a ranking; that is, for discretisation, the least important variable was chosen first, then another more relevant, and so on, until the top of a ranking was reached. On the right are included the charts for transformations going in descending order, starting with the most important features, and then those less and less relevant.

For the Naive Bayes classifier and the Relief ranking (see the charts in Figure 1), for the female writer dataset for both ascending and descending order, discretisation by unsupervised equal-frequency binning was noticeably advantageous; yet, in the vast majority of cases, when only some subset of the attributes was transformed instead of all of them. Only when proceeding upward and constructing seven or nine bins, or downward with two or seven intervals, did the maximum performance occur for all 12 discrete variables. For equal-width binning, the ascending order still resulted in benefits from partial discretisation, but less so, as for eight or ten bins, for discrete data, the predictions were only lower than the reference point in the continuous domain. For descending order, this reference point was higher than any classification accuracy obtained for partially or completely discretised sets for five out of nine data variants. Only when 5, 8, 9, or 10 intervals were constructed was there some small improvement, when only either two or three of the least important variables were transformed. When both the supervised discretisation methods were applied, for processing up the Relief ranking, enhanced predictions were detected for just three translated variables. Then, a very steep decrease followed. When the order of features was descending, only degradation of the classifier power was observed.

Recognition of the male writers with the NB classifier (see Figure 2) for variables transformed along the Relief ranking led to the conclusion that for ascending order, for all four discretisation methods, there was some improvement when a subset of variables was processed. For the unsupervised methods, only for duf03 and duw08 was the reference point in the continuous domain better than the results observed in partial discretisation. The descending order resulted in rather disappointing predictions for both supervised approaches, and for most duw versions of the data. Only for duw06 and duw07 could some benefits of discretisation be found. Equal-frequency binning returned slightly more advantageous cases, as for six out of a total of nine variants, the classifier working in the continuous domain was outperformed by the NB operating on partially discretised data.

The operation of the Naive Bayes classifier on the F-writer dataset discretised while following the OneR ranking is shown in the charts included in Figure 3. Discretisation using supervised methods for both ascending and descending orders of transformations resulted only in some gradually decreased predictions, dropping to very low levels when all attributes were translated. For unsupervised equal-frequency binning, for the ascending direction, when either 6, 7 or 9 bins were formed, the highest performance was reached only in the last processing step—when all variables were discrete. For the descending order, both reference points were the best for some variant of the data—for two bins constructed, discretisation brought only a decrease in predictions, and for seven intervals defined, when all features were transformed, the accuracy was the highest. For all the remaining cases, the maximum was found for partial discretisation. Unsupervised equal-width binning returned such data variants for which the results were poorer, but still some improvement was also noted. For upward direction and eight or ten bins, and for downward direction and three or six bins, the classifier working in the continuous domain was not outperformed in any other case, only degradation in power was observed. However, for other numbers of intervals constructed for variables, in both directions of processing along the OneR ranking, increased predictions were reported.

For the male writer dataset shown in Figure 4, in transformations following the OneR ranking, there were more instances where the NB inducer worked best in the continuous domain, in particular, for processing downward. From all the discretisation methods and their variants, only for equal-width binning with four or seven bins, or equal-frequency binning with 4, 5, 7, 9 or 10 intervals, was some enhanced accuracy observed, when only the first few most important attributes were discretised. On the other hand, for transformations starting with the least relevant variables, in all cases except one (for duw06), partial discretisation was advantageous to the classifier performance, for both supervised and unsupervised methods.

Figure 5 displays charts illustrating the performance for the J48 classifier when discretisation was executed while following the Relief ranking for the F-writer dataset. For some conditions, the transformation resulted only in either the same or worsened predictions. This happened for unsupervised equal-width binning with six bins for ascending order, and for descending, when for duf either 3, 5 or 9 intervals were constructed, and for duw with two or seven bins. The complete path of discretisation was beneficial in the case of duw08 while processing upward and, for dsK, for both directions. For other transformation conditions, translation of a subset of attributes resulted in the greatest advantage.

For the male writer dataset (see Figure 6), only in one instance were the predictions of the J48 inducer for the continuous variables the best, when unsupervised equal-width binning with two bins was applied starting with the highest ranking features. On the other end, the maximum accuracy for all discretised variables was detected for both supervised discretisation approaches and both directions of transformations, and also for duf03, duf07, duf09, and duf10 for going up the Relief ranking, and for going down for duf05, duf09, and for duw03. This leaves twelve other circumstances for ascending and fourteen for descending the ranking with some form of discretisation where partial transformation led to the greatest improvement.

For all discretisation variants driven by the OneR ranking, the performance trends of the J48 classifier are shown in Figure 7 for the cases where the female writers were recognised. Here, only for processing downward and unsupervised equal frequency and equal width with three intervals constructed, did discretisation cause worsened accuracy. For this dataset, the maximum performance was rarely observed for the translation of all attributes into a discrete domain, for the supervised Kononenko approach for both directions, and for duw08 for going upward. With the other transformation parameters, enhanced predictions were reported, yet typically for higher numbers of processed features when starting with the least ranked variables, or smaller numbers of attributes when a downward direction was considered.

When the samples were attributed to the male authors, which is shown in Figure 8, only unsupervised equal-width binning with two bins for descending OneR resulted in a decreased power of the J48 inducer. Discretisation of all variables was the most advantageous case when the equal-frequency binning approach with 2, 9 or 10 intervals was applied to the attributes processed up the ranking, and for five or ten bins when proceeding downward. For the Fayyad and Irani and the Kononenko supervised algorithms, in ascending and descending order, the maximum performance was reported in the last few steps or just the last step of the procedure. The remaining conditions, from all 20 discretisation paths, 13 for going up the ranking and 15 for going down, led to improved predictions when some groups of variables, but not all of them, were transformed.

How the k-NN classifier fared in the gradual discretisation procedure based on the Relief ranking for the female writers can be seen in Figure 9. Changing the domain from continuous to discrete for a subset or all variables was always beneficial, and the performance was improved at some point. An entirely discrete domain worked best with processing by the equal-frequency approach with 3, 4 or 6 bins while proceeding down the ranking, and for the equal-width algorithm with two intervals constructed for going down. For other numbers of intervals defined, only for subsets of variables in unsupervised algorithms for both directions of ordering were increased numbers of samples correctly attributed to authors. For supervised discretisation, the highest accuracy was detected after transformation of the three least relevant attributes, and for translating only the most important variable.

On the other hand, for the male writers, the trends in performance visible in Figure 10 were noticeably different. Discretisation brought many cases of worsened predictions. When starting the transformations with the least relevant attributes, this occurred for the duf algorithm applied with 2, 3, 6 or 8 bins, and for the duw approach for almost all numbers of intervals formed, with the exception of two and seven bins. For discretisation of the higher ranking variables first, the reference point in the continuous domain was also better than the results obtained for duf02, duf04, duf05, duf08, and duw02. The second reference point, with all attributes transformed, was the maximum for duf07 while processing up the Relief ranking, and for both supervised discretisation methods for proceeding down. This implies that for ascending direction in 8, and for descending order in 12, out of a total of 20 discretisation paths, the maximum was detected for partial transformations.

Figure 11 includes charts that allow for analysis of performance for the kNN classifier, when discretisation of the female writer dataset was based on the OneR ranking. For this ranking, the benefits of partial or complete discretisation can also be observed, as the obtained results were always better than in the continuous domain. Complete discretisation was the most advantageous in just a few instances; for the duf procedure with 3, 4, 6, and 8 bins for transformations starting with the least relevant features, and for the duw algorithm with two intervals when the highest ranking variables were translated first. Both supervised methods led to the maximum detected after only either the least or the most important attribute was discretised. For other discretisation conditions, the number of transformed features that led to the maximum predictions ranged from 1 to 7 for following the ascending ordering of variables, and from 1 to 10 for the reverse.

In contrast, for the male writers, discretisation conditioned on the OneR ranking brought (see Figure 12) similar observations to the Relief ranking referred to before, that is, rare cases of improvement over the performance in the continuous domain. For going up the ranking, partial discretisation gave the best results only for both supervised methods, and for duf02, duf04, and duf09. For proceeding down, the same happened for duf07, duf09, and duf10, as well as for almost all the duw variants, when four or more intervals were constructed. For the descending order, the supervised discretisation of all variables was most beneficial to author recognition.

Overall, these substantial experiments show a relatively high number of cases when transformation of domains from continuous to discrete, not for all but some selected variables, resulted in improved accuracy of the employed classifiers. This happened for all three inducers used in the research, for all discretisation approaches, both processing directions, and for both datasets. Finding which conditions were most advantageous requires further study, but the obtained results validate the research framework that was proposed and are sufficiently promising to provide motivation for deeper investigation.

### 5.3. Summary of Results

To evaluate the usefulness of the selective discretisation procedure, some standard statistics were calculated, as shown in Table 3, Table 4 and Table 5. They included the average classification accuracy and standard deviation (per sample), as well as the minimum and maximum performance observed. These elements were established based on the procedure with the starting point of a single variable out of *N* available attributes being discretised, and the last stage taken into consideration was when N−1 features became discrete. From the calculations, the performance in the original continuous domain, and the performance in the final discretisation step where all variables were in a discrete domain were excluded. For each discretisation method and each ranking, both directions, ascending (starting with less important variables) and descending (starting at the top of a ranking with the most relevant features), were considered for each classifier. For the unsupervised methods, the results include detailed values obtained for each variant of a method (depending on the number of bins constructed for the variables), the overall averages calculated for the approach, and the overall extrema as well.

From these statistics, the highest values were preferred for all but one. For the standard deviation, the smallest values show how stable the process was, that the obtained predictions were close to each other. For classification accuracy, whether for averages calculated or extrema found, a higher percentage of correctly attributed samples was considered advantageous, as always in classification tasks. These preferred minimal and maximal values were marked in the tables in the context of each ranking and direction of processing for a dataset and a classifier.

In all three tables, it was observed that the standard deviation had values over a relatively wide range. There were always some cases of double digits in the integer parts. They were calculated for the supervised discretisation algorithms applied to the attributes, either one or both, and this happened for both ranking directions and both rankings. For the unsupervised discretisation approaches, the results were smaller, in single digits, and quite often just fractional. The minimum value was always found for one of the unsupervised transformations of the variables.

For the Naive Bayes classifier, the statistics presented in Table 3 show that, for the most part, for both rankings studied, for the female writer dataset, discretisation brought improved performance. For the male writer dataset, there were many cases of degradation when just the average was studied. For unsupervised equal-frequency binning and F-writers, in the vast majority of cases, the average performance was better than the one reported in the input continuous domain. However, for M-writers, the predictions decreased, although the change was mostly small. The best results were rarely observed for unsupervised equal-width binning, and, surprisingly, also for supervised discretisation methods. The maximum level of predictions for the female writer dataset was the same for both rankings, and for ascending order, the same as the highest performance detected for all variables transformed at once. For the male writer dataset, the maximum classification accuracy was always higher than the performance in the continuous domain and than for all discretised variables.

Comparison of the two orderings of variables in this case leads to the conclusion that the Relief ranking processed in the ascending direction for the female writers brought better results than the OneR, while for the male writers the opposite was true. Comparison of the directions indicates that going upward was more advantageous than following a ranking downward.

The J48 inducer (see Table 4) was generally not as good at prediction as Naive Bayes. It benefited more from discretisation. For the ascending direction of the Relief and OneR rankings, the average performance was almost always better than in the continuous domain for both the female and male writer datasets. For the descending direction of both rankings, these values showed some decrease with respect to the reference point. The maximum predictions found for the male writers were higher than the best result obtained for all discretised variables. For the female writers, the same situation occurred for the Relief ranking processed in both directions, and for ascending order, only Relief led to higher precision.

Of the three classifiers studied, k-NN (see Table 5) returned the worst predictions for the female writers, lower than the other two inducers. When only some parts of attributes were discretised, for the ascending direction of processing a ranking, the average performance was lower than when all variables were transformed. For the descending direction, there were some cases of improvement. However, the maximum classification accuracy detected was always higher, both for the rankings and in both directions. For the male writers, the average performance was close, yet below the reference point in the continuous domain, and noticeably below the predictions for all discrete attributes. The maximum found was improved only when processing a ranking up.

When the results from all three tables were compared against each other, it turned out that for the female writer dataset, ascending orders for rankings were beneficial for classification by the Naive Bayes and J48, while for the k-NN, the opposite was true. For the male writer dataset, the trends were not so constant and depended on a classifier and a discretisation approach applied to the data. However, they were the same for both rankings employed in the research. For F-writers, it was also observed that the Relief ranking more often led to higher values of the obtained statistics than the OneR ranking, while for M-writers, more variations were detected and greater dependence on the parameters of the discretisation process observed.

For the process of gradual discretisation controlled by the importance of attributes indicated by a ranking, the detailed analysis of classifier performance indicates that standard transformation approaches cannot necessarily guarantee a form of features that is the most beneficial to predictions. When only some subsets of variables are discretised, instead of all of them at once, it can lead to improved accuracy. Many such cases were observed in the investigations, for all types of inducers employed and all variants of discrete data. These findings show the merits of the proposed methodology, although they also indicate that finding the most advantageous scenario, that is, a particular discretisation method and a subset of variables to transform, is not a trivial task. To limit computational costs, the processing does not have to include all steps, from discretisation of a single attribute to translation of all of them. It can be stopped sooner, once some increase in performance is detected. However, such an approach brings the risk of detecting only a local (and not global) maximum.

## 6. Conclusions

Discretisation as a stage of data preparation plays an important role in knowledge discovery processes, with a noticeable impact on their efficiency. In standard proceedings, some arbitrarily selected discretisation approach, which enables finding categorical representations for the continuous domains of the input features, is applied to all attributes present in the input data, regardless of their characteristics, and all transformations are performed at once. In the paper, the research methodology was presented which was dedicated to a gradual data discretisation procedure, driven by a ranking of attributes. The aim was to examine how the form of the features, dependent on their importance, affects their characteristics and impacts the performance of the chosen classifiers.

Rankings belong to feature selection mechanisms. They provide information on the importance of individual features, which allows reducing the dimensionality of the data. In the research, the constructed orderings of variables were exploited to direct the sequential transformations of attributes. The features were selected one-by-one, taking into account two possible directions of processing, i.e., descending and ascending. The former started with the most relevant attributes and then gradually less and less important variables were discretised, and the latter began at the bottom of a ranking with the least relevant features before moving on to the more important ones. For transformations, representatives of the two most popular approaches were used: supervised and unsupervised.

The research methodology was extensively verified on two collections of the datasets from the stylometry domain, i.e., on 901 variants per one collection in total. These investigations included four discretisation algorithms with various parameters, two ranking methods with both directions of ordering of features, and three state-of-the-art classifiers. The analysis of the results obtained allowed the identification of many cases where the proposed data transformation procedure resulted in an improved predictive accuracy for the classifiers, when only some subset of attributes was discretised. This proves the merits of the methodology and highlights the value of investigating it more deeply.

In future research, other discretisation algorithms and classifiers will be examined, with the goal of determining guidelines for selecting the most advantageous combinations of attribute transformation methods. Also, other mechanisms for ranking construction will be investigated and compared.

## Figures and Tables

**Table 1:** Rankings of attributes.

Rank	F-Writers			M-Writers	
	Relief	OneR		Relief	OneR
1	on	to		by	by
2	to	on		if	or
3	of	of		so	in
4	as	as		or	if
5	by	by		in	at
6	if	if		as	so
7	or	in		at	as
8	up	up		on	on
9	at	so		no	no
10	in	or		of	to
11	so	at		up	of
12	no	no		to	up

**Table 2:** Performance [%] of classifiers for all attributes in the continuous domain and all discrete domains.

Domain	F-Writers				M-Writers			Domain	F-Writers				M-Writers		
	NB	J48	k-NN		NB	J48	k-NN		NB	J48	k-NN		NB	J48	k-NN
Cont.	93.33	89.79	85.56		**84.03**	75.63	77.29								
dsF	50.00	87.78	62.22		69.44	80.76	**83.54**	dsK	62.22	**93.40**	62.22		68.89	80.76	82.43
duf02	91.18	91.18	83.89		75.35	73.82	68.47	duw02	87.01	85.35	88.06		73.82	72.71	70.14
duf03	92.85	85.63	86.94		75.69	79.72	71.25	duw03	89.38	83.68	85.83		78.68	77.29	72.36
duf04	93.40	86.46	88.61		81.60	69.31	75.21	duw04	91.67	86.94	82.78		80.63	79.24	71.32
duf05	92.85	86.18	85.07		81.11	78.96	73.54	duw05	90.56	80.42	84.10		79.38	75.76	69.51
duf06	94.10	88.19	**89.17**		80.35	74.79	73.47	duw06	92.22	85.69	85.21		82.22	75.21	77.22
duf07	**95.76**	81.74	85.63		80.42	78.75	77.85	duw07	91.11	89.65	86.46		81.04	71.94	72.29
duf08	92.29	88.19	88.47		80.49	77.92	75.28	duw08	91.67	90.97	85.76		77.64	75.42	73.61
duf09	94.58	88.26	86.81		80.42	79.79	77.57	duw09	90.56	90.35	88.68		81.53	76.46	74.58
duf10	93.47	87.15	86.88		79.86	**80.90**	75.35	duw10	91.67	87.01	86.32		80.00	76.25	73.61

**Table 3:** Statistics of performance [%]: average classification accuracy, standard deviation, and minimum and maximum classification accuracy of the NB classifiers, for the procedure of the gradual discretisation controlled by rankings, starting with 1 out of *N* discretised attributes, and ending with N−1 discretised variables.

	F-Writers						M-Writers					
	**Ascending**			**Descending**			**Ascending**			**Descending**		
	**Avg. ± St.dev.**	**Min**	**Max**	**Avg. ± St.dev.**	**Min**	**Max**	**Avg. ± St.dev.**	**Min**	**Max**	**Avg. ± St.dev.**	**Min**	**Max**
Domain	Relief ranking											
dsF	80.38 ± 12.94	56.18	95.14	60.18 ± 14.52	50.00	88.61	83.74 ± 1.79	81.53	**87.08**	76.60 ± 4.13	72.29	82.85
dsK	83.67 ± 07.50	74.17	95.14	78.57 ± 11.70	61.11	90.56	83.15 ± 1.29	81.32	84.79	75.96 ± 4.71	70.83	82.85
duf	94.07 ± 00.19	92.71	95.76	93.00 ± 00.49	89.38	95.21	82.73 ± 1.80	77.43	85.21	80.95 ± 1.84	73.13	85.76
duf02	**94.58** ± 00.58	**93.89**	**95.76**	90.16 ± 00.60	89.38	91.11	81.98 ± 2.33	77.43	84.51	77.14 ± 3.64	73.13	83.54
duf03	94.35 ± 00.50	**93.89**	95.21	93.20 ± 00.98	91.67	94.58	82.64 ± 1.49	79.93	84.03	79.07 ± 3.28	74.03	83.40
duf04	94.34 ± 00.63	**93.89**	**95.76**	93.16 ± 00.81	92.29	94.51	83.13 ± 2.12	79.93	85.21	81.76 ± 1.47	79.79	84.65
duf05	93.67 ± 00.40	92.71	93.89	92.34 ± 01.00	90.49	93.96	82.68 ± 1.93	79.24	84.65	**82.96** ± 1.69	80.56	85.21
duf06	93.95 ± 00.32	93.33	94.51	93.54 ± 00.47	92.85	94.51	82.80 ± 2.00	79.38	85.21	80.90 ± 1.22	79.79	83.40
duf07	94.12 ± 00.49	93.33	95.14	**94.53** ± **00.44**	**93.33**	**95.21**	82.69 ± 1.85	79.93	85.14	81.52 ± 1.61	79.24	84.65
duf08	93.73 ± 00.38	92.71	93.89	93.11 ± 00.94	91.74	95.07	82.60 ± 1.93	79.31	85.21	81.87 ± 1.69	80.35	84.58
duf09	93.90 ± **00.27**	93.33	94.51	94.00 ± 00.57	**93.33**	95.14	82.73 ± 1.92	79.86	85.21	82.14 ± 1.43	80.42	84.58
duf10	94.00 ± 00.38	**93.89**	95.14	93.01 ± 00.82	92.29	94.51	83.33 ± 1.44	80.49	85.21	81.18 ± 1.88	79.24	**85.76**
duw	93.15 ± 00.61	90.69	95.76	91.12 ± 00.97	85.21	93.89	83.30 ± 1.11	79.86	86.46	81.01 ± 0.92	76.88	84.17
duw02	93.88 ± 00.92	92.22	**95.76**	88.17 ± 02.26	85.21	92.22	83.67 ± **0.79**	**82.71**	85.14	78.87 ± 1.19	77.29	80.76
duw03	92.99 ± 01.05	91.04	94.51	89.64 ± 01.22	88.13	92.08	83.42 ± 2.03	79.93	86.46	79.65 ± 1.89	76.88	82.85
duw04	93.24 ± 00.66	91.67	94.03	91.46 ± 00.84	90.56	93.33	82.66 ± 1.26	80.00	84.51	81.75 ± 1.09	80.00	83.96
duw05	92.84 ± 00.94	90.69	93.89	91.18 ± 01.60	89.38	93.89	83.24 ± 2.05	79.86	85.28	81.11 ± 1.00	79.86	83.47
duw06	93.25 ± 00.54	91.74	93.96	92.17 ± 00.87	91.04	93.33	83.48 ± 1.09	81.04	84.58	81.22 ± 1.32	79.44	84.10
duw07	93.05 ± 00.86	91.11	94.03	92.01 ± 00.52	91.53	92.78	83.40 ± 1.03	80.49	84.51	82.59 ± **0.90**	**81.11**	84.17
duw08	93.08 ± 00.46	92.22	93.33	92.08 ± 00.87	91.04	93.89	82.38 ± 1.26	79.93	83.96	80.64 ± 1.47	78.75	82.85
duw09	92.89 ± 00.59	91.67	93.89	91.28 ± 01.28	89.93	93.26	83.43 ± 1.10	81.53	84.58	81.72 ± 0.83	80.49	83.47
duw10	93.09 ± 00.67	91.11	93.33	92.07 ± 01.06	91.11	93.89	**83.99** ± 1.21	81.67	85.76	81.55 ± 1.00	79.38	82.85
	OneR ranking											
dsF	78.54 ± 12.29	52.22	93.33	60.61 ± 15.34	50.00	92.78	**84.32** ± 1.58	81.46	**87.08**	76.06 ± 3.87	71.67	83.33
dsK	81.07 ± 08.04	61.88	93.33	82.92 ± 08.32	61.11	92.78	83.59 ± 0.94	**81.94**	84.72	75.56 ± 4.49	68.33	83.33
duf	93.88 ± 00.19	92.22	95.76	93.14 ± 00.51	89.38	95.21	83.09 ± 1.60	79.24	85.28	80.81 ± 1.73	73.06	85.76
duf02	**94.42** ± 00.73	93.33	**95.76**	90.33 ± 01.12	89.38	93.33	82.47 ± 1.64	79.65	84.51	77.28 ± 3.84	73.06	83.54
duf03	94.02 ± 00.44	93.47	95.14	**93.54** ± 00.73	92.22	94.58	82.78 ± 1.67	79.93	84.65	78.00 ± 3.24	74.03	83.40
duf04	94.02 ± 00.45	93.40	95.14	93.43 ± 00.79	92.29	94.51	83.54 ± 1.68	80.42	85.28	81.76 ± 1.37	79.79	84.65
duf05	93.52 ± 00.58	92.22	93.89	92.23 ± 01.26	90.49	93.96	83.25 ± 1.92	79.24	84.72	**82.80** ± 1.46	81.04	84.58
duf06	93.90 ± **00.04**	**93.89**	94.03	93.50 ± 00.73	92.29	94.51	83.43 ± 1.44	80.42	85.21	81.22 ± 1.36	79.79	83.47
duf07	93.91 ± 00.27	93.33	94.51	94.80 ± **00.44**	**93.89**	**95.21**	82.68 ± 1.80	79.93	85.14	81.35 ± 1.64	79.24	84.65
duf08	93.63 ± 00.59	92.22	93.89	93.15 ± 01.19	91.67	95.07	83.14 ± 1.35	80.49	85.21	81.70 ± 1.17	80.35	83.96
duf09	93.74 ± 00.34	92.85	93.89	94.01 ± 00.55	93.47	95.14	83.06 ± 1.95	79.86	85.21	81.76 ± 1.22	80.42	84.58
duf10	93.79 ± 00.31	92.85	93.89	93.29 ± 00.70	92.29	94.51	83.42 ± 1.57	80.49	85.21	81.44 ± 1.83	79.24	**85.76**
duw	93.11 ± 00.81	89.44	95.76	91.42 ± 01.04	86.39	94.51	83.05 ± 1.19	79.86	86.46	81.11 ± 1.03	74.38	84.17
duw02	93.51 ± 01.52	89.44	**95.76**	88.83 ± 02.21	86.39	93.89	83.32 ± 1.34	80.90	85.14	78.48 ± 1.90	74.38	80.63
duw03	92.94 ± 01.11	90.63	93.89	90.25 ± 01.30	88.13	92.78	83.91 ± 1.71	79.93	86.46	80.16 ± 1.75	77.50	82.85
duw04	93.39 ± 00.90	91.04	94.03	91.20 ± 01.14	89.93	93.89	82.18 ± 1.57	80.35	84.51	82.17 ± 1.18	**81.18**	84.10
duw05	92.82 ± 00.99	90.49	93.89	91.69 ± 01.54	89.38	93.89	83.15 ± 1.81	79.86	85.28	81.25 ± 1.60	78.75	83.47
duw06	93.24 ± 00.58	91.60	93.96	92.27 ± 00.83	91.04	93.33	83.37 ± **0.88**	81.04	84.03	81.37 ± **0.84**	80.00	82.36
duw07	93.10 ± 00.74	91.67	94.03	92.49 ± 00.63	91.67	93.89	83.30 ± 1.26	80.49	84.65	82.27 ± 1.04	81.04	84.17
duw08	93.08 ± 00.46	92.22	93.33	92.17 ± 00.92	91.04	93.89	81.79 ± 1.86	79.86	84.58	80.56 ± 1.75	77.64	83.47
duw09	92.97 ± 00.85	91.04	93.89	91.54 ± 01.48	89.93	94.51	83.11 ± 1.26	81.53	84.58	81.81 ± 0.86	80.56	83.47
duw10	92.93 ± 00.90	91.11	93.33	92.32 ± 00.96	91.11	93.89	83.30 ± 1.18	81.60	84.58	81.94 ± 1.04	80.00	83.47

**Table 4:** Statistics of performance [%]: average classification accuracy, standard deviation, and minimum and maximum classification accuracy of the J48 classifiers, for the procedure of the gradual discretisation controlled by the rankings, starting with 1 out of *N* discretised attributes, and ending with N−1 discretised variables.

	F-Writers						M-Writers					
	**Ascending**			**Descending**			**Ascending**			**Descending**		
	**Avg. ± St.dev.**	**Min**	**Max**	**Avg. ± St.dev.**	**Min**	**Max**	**Avg. ± St.dev.**	**Min**	**Max**	**Avg. ± St.dev.**	**Min**	**Max**
Domain	Relief ranking											
dsF	**91.45** ± 1.57	**89.79**	**94.65**	74.36 ± 19.08	50.00	90.28	77.53 ± 2.36	72.01	79.51	**78.59** ± 1.64	**76.32**	80.76
dsK	91.16 ± 1.17	**89.79**	92.78	**92.41** ± 01.93	87.50	93.40	76.76 ± 2.69	71.88	80.14	**78.59** ± 1.64	**76.32**	80.76
duf	89.95 ± 0.92	82.64	93.33	87.54 ± 00.73	82.85	92.22	76.37 ± 0.71	70.83	80.90	74.77 ± 0.81	66.67	82.08
duf02	90.55 ± 1.77	85.69	92.78	88.43 ± 02.42	84.79	91.81	73.64 ± 1.84	72.01	77.85	71.87 ± 3.92	66.74	77.92
duf03	90.24 ± 1.48	86.81	93.33	85.73 ± 00.58	85.28	87.43	76.52 ± 1.31	74.44	78.89	77.54 ± 2.48	72.15	81.46
duf04	90.38 ± 0.82	89.24	91.53	89.36 ± 02.56	86.46	92.22	77.07 ± 1.29	**75.63**	79.58	72.97 ± 4.56	66.67	79.51
duf05	89.35 ± 2.36	83.47	92.78	84.68 ± 01.01	83.75	87.01	**78.43** ± 1.54	**75.63**	80.14	71.27 ± **0.82**	69.86	72.64
duf06	88.77 ± 2.75	82.64	90.42	90.75 ± 01.15	88.19	91.88	75.13 ± 1.99	70.83	77.92	75.00 ± 1.57	72.22	77.43
duf07	90.34 ± 0.76	**89.79**	92.15	84.36 ± 02.42	82.85	90.49	75.64 ± **0.74**	74.58	76.88	78.05 ± 2.14	73.26	80.42
duf08	90.32 ± 0.60	**89.79**	91.53	88.42 ± 00.67	87.64	90.00	77.54 ± 2.57	73.89	80.90	76.84 ± 1.90	72.64	78.47
duf09	90.13 ± **0.41**	**89.79**	90.97	88.09 ± 00.57	86.53	88.33	77.51 ± 1.01	**75.63**	78.75	74.43 ± 2.79	69.65	79.79
duf10	89.48 ± 0.91	86.88	90.35	88.04 ± 01.27	87.15	91.81	75.81 ± 1.13	72.92	77.36	74.94 ± 4.14	69.38	**82.08**
duw	89.83 ± 1.22	83.68	92.85	87.63 ± 00.83	80.35	94.51	76.73 ± 1.13	68.13	81.32	74.42 ± 0.98	63.19	79.86
duw02	90.73 ± 1.19	88.61	92.22	84.22 ± 03.09	80.35	89.38	76.67 ± 1.57	73.68	78.96	69.15 ± 3.70	63.19	74.51
duw03	89.79 ± 2.30	83.68	92.78	87.51 ± 01.89	83.68	89.93	76.87 ± 2.02	72.43	80.14	73.81 ± 2.81	68.82	77.29
duw04	90.61 ± 1.20	88.54	92.22	86.81 ± 01.81	83.26	90.97	77.90 ± 2.09	74.10	**81.32**	76.50 ± 2.45	71.11	79.86
duw05	89.83 ± 2.27	85.49	92.22	85.07 ± 02.45	80.42	89.93	76.84 ± 2.00	74.79	80.69	74.96 ± 1.37	72.78	77.43
duw06	89.03 ± 1.19	86.25	89.79	86.12 ± 01.34	85.00	89.93	74.68 ± 1.75	72.50	76.88	74.05 ± 1.71	71.60	76.39
duw07	89.98 ± 1.55	85.90	92.15	89.46 ± **00.49**	88.06	89.65	76.87 ± 1.65	74.58	79.10	74.44 ± 1.67	70.00	76.04
duw08	89.44 ± 1.86	84.51	90.42	90.81 ± 01.73	86.94	92.22	76.69 ± 3.66	68.13	79.51	75.43 ± 1.64	72.50	79.44
duw09	89.20 ± 1.56	85.00	90.42	91.16 ± 01.45	**90.35**	**94.51**	77.15 ± 1.56	75.35	79.58	74.48 ± 2.18	71.18	77.43
duw10	89.82 ± 1.79	85.14	92.85	87.56 ± 01.44	87.01	91.74	76.86 ± 1.63	74.93	78.96	76.98 ± 1.26	74.03	78.61
	OneR ranking											
dsF	89.46 ± 6.83	69.17	92.78	63.62 ± 18.74	50.00	90.28	77.77 ± 1.71	74.38	79.51	**78.52** ± 2.04	74.10	80.76
dsK	90.65 ± 3.01	82.29	92.78	**91.98** ± 03.22	82.78	93.40	77.04 ± 1.52	74.24	79.51	**78.52** ± 2.04	74.10	80.76
duf	89.91 ± 1.27	82.64	93.33	87.66 ± 01.01	82.85	92.22	76.57 ± 0.91	71.88	82.08	74.32 ± 0.60	66.67	81.46
duf02	**90.99** ± 0.85	**89.79**	92.78	89.00 ± 02.61	84.79	91.81	74.72 ± 2.14	71.88	78.40	71.71 ± 4.34	66.74	79.10
duf03	90.13 ± 1.77	85.63	**93.33**	85.81 ± 00.58	85.28	87.43	77.47 ± 2.25	74.79	**82.08**	77.23 ± 1.73	**74.51**	**81.46**
duf04	90.16 ± 1.68	85.63	91.53	89.36 ± 02.33	86.46	91.81	78.33 ± 1.90	76.60	81.94	71.95 ± 3.34	66.67	77.71
duf05	89.89 ± 1.55	86.94	92.78	85.01 ± 02.18	83.75	90.97	78.02 ± 1.09	**76.67**	80.14	72.22 ± 2.02	70.00	76.18
duf06	89.07 ± 2.61	82.64	90.42	89.73 ± 01.85	86.25	91.88	75.62 ± 1.54	73.54	77.92	74.20 ± 1.45	72.22	76.53
duf07	89.88 ± 2.40	82.92	92.15	84.63 ± 02.82	82.85	92.22	75.60 ± **0.88**	74.58	76.88	77.30 ± 2.67	73.26	80.42
duf08	89.97 ± 1.12	87.08	91.53	88.83 ± 01.26	88.19	92.15	76.46 ± 1.55	73.89	78.54	76.58 ± 2.11	72.64	79.10
duf09	89.85 ± 1.06	86.88	90.97	88.34 ± 00.87	86.53	90.42	77.66 ± 0.94	76.11	78.47	73.54 ± 2.57	69.65	77.50
duf10	89.20 ± 1.34	86.25	90.35	88.22 ± 01.50	87.15	91.81	75.28 ± 1.21	72.92	77.36	74.14 ± 3.75	69.38	79.31
duw	90.09 ± 0.88	82.22	92.85	87.63 ± 01.31	80.35	94.51	77.18 ± 1.35	68.13	81.32	74.37 ± 0.65	63.89	79.79
duw02	90.59 ± 1.72	87.01	92.22	84.68 ± 03.50	80.35	90.35	76.63 ± 3.25	70.69	79.72	68.74 ± 2.48	63.89	72.22
duw03	90.73 ± 1.13	89.24	92.78	86.73 ± 02.35	83.06	89.17	**78.39** ± 1.63	76.25	80.83	74.57 ± 2.52	68.82	77.36
duw04	90.20 ± 2.26	84.58	92.22	87.45 ± 01.68	85.14	90.97	77.58 ± 2.41	73.26	81.32	76.20 ± 2.66	71.11	79.79
duw05	90.64 ± 2.04	85.49	92.22	84.57 ± 03.80	80.42	92.22	78.11 ± 1.96	74.24	80.69	74.72 ± 1.56	72.78	77.99
duw06	89.24 ± **0.78**	87.92	90.35	85.84 ± 01.66	84.51	90.35	75.20 ± 1.79	72.01	77.85	74.59 ± **0.82**	73.40	76.25
duw07	90.39 ± 0.89	88.54	92.15	89.67 ± **00.30**	89.10	90.42	76.13 ± 2.19	73.61	79.10	73.56 ± 2.33	70.28	76.04
duw08	88.80 ± 2.75	82.22	90.42	91.07 ± 01.24	88.06	92.22	77.67 ± 3.27	68.13	79.51	75.66 ± 1.93	72.85	79.44
duw09	89.75 ± 0.82	88.06	90.42	91.05 ± 01.55	**89.79**	**94.51**	77.36 ± 1.88	74.79	79.58	75.39 ± 1.97	73.06	78.26
duw10	90.47 ± 1.08	**89.79**	92.85	87.59 ± 01.46	87.01	91.74	77.56 ± 1.39	74.93	79.51	75.94 ± 1.64	73.06	78.61

**Table 5:** Statistics of performance [%]: average classification accuracy, standard deviation, and minimum and maximum classification accuracy of the k-NN classifiers, for the procedure of the gradual discretisation controlled by the rankings, starting with 1 out of *N* discretised attributes, and ending with N−1 discretised variables.

	F-Writers						M-Writers					
	**Ascending**			**Descending**			**Ascending**			**Descending**		
	**Avg. ± St.dev.**	**Min**	**Max**	**Avg. ± St.dev.**	**Min**	**Max**	**Avg. ± St.dev.**	**Min**	**Max**	**Avg. ± St.dev.**	**Min**	**Max**
Domain	Relief ranking											
dsF	72.85 ± 11.39	57.78	**91.04**	64.47 ± 09.61	55.56	85.83	77.32 ± 4.10	70.69	**85.76**	75.26 ± 2.86	70.14	78.61
dsK	74.06 ± 10.11	66.67	**91.04**	71.43 ± 09.23	57.22	85.83	**77.83** ± 4.04	69.58	85.21	75.41 ± 3.04	70.14	79.17
duf	83.80 ± 02.53	73.89	88.75	88.79 ± 01.11	81.94	93.47	72.24 ± 1.74	61.81	78.68	74.12 ± 0.94	64.72	80.00
duf02	81.48 ± 05.85	73.89	88.68	86.36 ± 02.20	81.94	89.72	67.80 ± 5.48	61.94	76.04	71.70 ± 1.69	69.31	74.58
duf03	81.24 ± 02.80	76.32	86.88	89.49 ± 01.76	86.88	**93.47**	67.69 ± 4.17	61.81	73.61	73.24 ± 3.14	68.96	80.00
duf04	83.74 ± 02.73	79.17	87.36	90.08 ± 01.20	88.68	92.78	73.28 ± 2.64	69.79	78.68	74.65 ± 1.80	70.90	76.46
duf05	84.68 ± 02.96	79.72	88.61	87.84 ± 02.35	84.65	92.01	72.30 ± 2.85	69.24	77.71	71.45 ± 3.84	64.72	77.08
duf06	85.14 ± 01.25	82.57	86.88	**90.43** ± 00.84	**88.75**	91.60	74.13 ± 2.46	70.49	77.22	74.76 ± 2.31	70.35	77.64
duf07	82.95 ± 03.16	78.68	86.88	87.90 ± 01.57	85.83	90.97	73.48 ± 2.37	70.14	77.78	76.75 ± 1.63	74.58	79.51
duf08	85.28 ± 02.92	80.35	88.75	88.76 ± 01.38	87.36	91.39	73.28 ± 1.92	70.14	75.49	73.52 ± 1.73	70.97	76.32
duf09	84.96 ± 02.28	80.90	87.43	89.04 ± 01.47	86.81	90.90	74.73 ± 1.35	**73.68**	78.40	75.74 ± 1.48	73.68	78.19
duf10	84.75 ± 02.38	80.90	87.50	89.20 ± 01.56	86.39	91.53	73.45 ± 1.65	71.04	75.83	75.29 ± 2.09	71.74	79.31
duw	86.09 ± 01.75	73.82	90.97	86.47 ± 01.20	80.35	90.83	72.80 ± 1.06	65.90	80.07	75.25 ± 1.81	63.06	82.50
duw02	85.94 ± 03.10	79.79	89.93	83.06 ± 01.45	80.35	85.07	73.40 ± 4.38	66.67	80.07	70.16 ± 4.49	63.06	77.08
duw03	82.17 ± 03.79	73.82	86.94	86.86 ± 02.93	82.64	90.83	71.00 ± 1.72	68.19	74.72	74.07 ± 2.57	70.49	78.47
duw04	86.45 ± 02.19	82.78	89.10	85.46 ± 02.49	82.71	89.79	73.62 ± 2.69	68.47	76.53	76.00 ± 2.19	71.67	78.33
duw05	86.40 ± 02.36	82.36	89.17	85.93 ± 02.38	83.40	89.79	68.87 ± 1.75	65.90	71.46	74.21 ± 2.86	70.28	78.47
duw06	86.07 ± 01.70	82.36	88.06	86.75 ± 02.18	83.96	89.58	74.03 ± 1.67	70.63	76.04	77.47 ± **1.27**	**75.35**	80.69
duw07	87.18 ± 01.99	85.07	90.97	87.51 ± **00.75**	86.39	88.61	74.74 ± 2.43	70.56	77.71	74.29 ± 2.00	71.32	77.50
duw08	86.22 ± 01.87	82.22	88.54	87.02 ± 01.18	84.51	88.54	73.35 ± 2.05	69.58	76.74	78.02 ± 2.33	75.00	**82.50**
duw09	**87.76** ± **01.30**	**85.14**	89.79	89.06 ± 01.05	86.81	90.42	73.51 ± 1.50	71.53	76.46	74.63 ± 2.38	70.83	79.58
duw10	86.62 ± 02.11	83.47	89.17	86.55 ± 00.98	84.58	88.47	72.67 ± **1.30**	70.76	74.38	**78.36** ± 1.95	74.79	81.53
	OneR ranking											
dsF	68.31 ± 07.42	57.78	88.13	66.05 ± 10.03	58.33	90.28	76.89 ± 3.17	**74.03**	**85.76**	75.76 ± 3.74	67.01	**81.25**
dsK	69.48 ± 06.28	66.11	88.13	73.74 ± 07.95	60.56	90.28	**77.92** ± 3.68	73.47	85.21	75.86 ± 3.80	67.01	**81.25**
duf	83.98 ± 02.64	73.89	89.24	89.22 ± 00.83	83.26	93.47	72.57 ± 1.70	61.04	79.38	73.65 ± 0.90	64.72	78.82
duf02	80.65 ± 05.21	73.89	87.36	86.48 ± 02.08	83.26	90.28	68.53 ± 6.00	61.04	78.96	72.75 ± 3.46	66.18	77.50
duf03	82.22 ± 03.29	76.32	86.88	89.07 ± 02.15	86.39	**93.47**	68.63 ± 2.72	63.47	72.15	70.54 ± 2.52	65.56	75.07
duf04	84.60 ± 03.20	79.17	88.19	90.10 ± 01.26	87.99	92.01	73.51 ± 2.21	70.28	77.64	75.73 ± **1.24**	72.78	77.01
duf05	84.63 ± 03.38	79.72	89.24	88.55 ± 01.72	85.14	92.01	71.45 ± 1.48	69.24	74.44	71.43 ± 3.50	64.72	75.90
duf06	85.93 ± 01.61	82.57	88.61	**90.43** ± 00.87	88.54	91.60	74.37 ± 2.23	71.39	77.22	73.91 ± 2.00	70.97	77.15
duf07	82.75 ± 02.87	78.68	86.88	88.63 ± 01.45	86.18	90.97	74.27 ± 2.57	71.32	79.38	75.96 ± 2.22	71.18	78.82
duf08	85.25 ± 02.85	80.35	87.99	89.70 ± 01.17	87.36	91.39	73.74 ± 2.04	70.14	75.97	73.14 ± 1.65	70.69	75.76
duf09	85.32 ± 02.43	80.90	87.99	89.79 ± 01.37	87.22	91.46	75.13 ± 1.70	73.13	77.78	74.76 ± 2.30	70.56	77.71
duf10	84.50 ± 02.29	80.90	88.54	90.24 ± 00.98	**88.61**	91.60	73.50 ± 1.91	71.04	77.15	74.65 ± 2.07	71.74	78.19
duw	85.73 ± 01.68	73.82	90.97	86.98 ± 01.00	80.35	90.21	72.53 ± 1.56	64.65	77.29	74.61 ± 1.92	66.74	80.35
duw02	85.12 ± 03.53	77.43	88.68	84.17 ± 01.91	80.35	87.92	72.60 ± 4.13	64.65	77.01	72.76 ± 3.76	67.71	77.22
duw03	81.81 ± 03.64	73.82	86.25	87.08 ± 02.31	82.64	90.21	72.52 ± 1.60	70.21	75.14	72.96 ± 2.51	67.78	76.88
duw04	86.07 ± 02.35	81.11	88.61	86.12 ± 02.19	82.85	89.79	73.04 ± 1.65	70.35	75.56	73.98 ± 2.52	69.17	77.78
duw05	85.42 ± 02.04	82.36	89.17	86.71 ± 02.23	84.10	89.79	69.29 ± 2.09	65.90	74.17	72.58 ± 3.12	66.74	77.92
duw06	86.19 ± **01.57**	82.36	87.43	87.65 ± 01.50	85.28	89.58	74.10 ± 1.99	70.63	77.29	75.80 ± 2.56	69.51	77.64
duw07	87.34 ± 02.12	85.00	**90.97**	88.18 ± **00.79**	87.01	89.93	73.36 ± 1.81	70.56	75.83	74.19 ± 2.17	71.04	78.33
duw08	86.22 ± 02.04	82.22	88.68	87.60 ± 00.82	86.25	88.54	72.92 ± 2.87	69.58	77.22	77.05 ± 1.74	**74.38**	79.72
duw09	**87.62** ± 01.61	**85.14**	89.79	88.58 ± 00.91	86.88	89.72	72.78 ± 1.73	71.11	75.90	74.53 ± 3.14	68.61	79.58
duw10	85.74 ± 01.64	83.47	88.06	86.70 ± 01.15	84.58	88.47	72.16 ± **1.40**	70.00	74.38	**77.62** ± 1.98	73.54	80.35

## Data Availability

Data available upon request.
